# Surface-Area-to-Volume Ratio Determines Surface Tensions in Microscopic, Surfactant-Containing Droplets

**DOI:** 10.1021/acscentsci.3c00998

**Published:** 2023-10-24

**Authors:** Alison Bain, Kunal Ghosh, Nønne L. Prisle, Bryan R. Bzdek

**Affiliations:** † School of Chemistry, University of Bristol, Bristol BS8 1TS, United Kingdom; ‡ Center for Atmospheric Research, University of Oulu, Oulu 90014, Finland

## Abstract

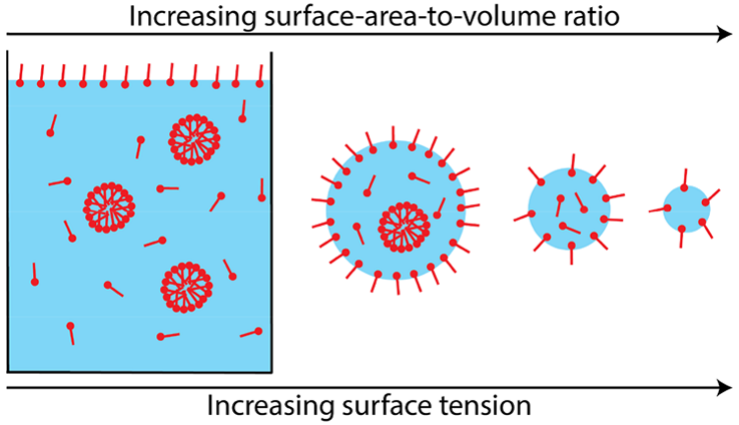

The surface composition of aerosol droplets is central to predicting cloud droplet number concentrations, understanding atmospheric pollutant transformation, and interpreting observations of accelerated droplet chemistry. Due to the large surface-area-to-volume ratios of aerosol droplets, adsorption of surfactant at the air–liquid interface can deplete the droplet’s bulk concentration, leading to droplet surface compositions that do not match those of the solutions that produced them. Through direct measurements of individual surfactant-containing, micrometer-sized droplet surface tensions, and fully independent predictive thermodynamic modeling of droplet surface tension, we demonstrate that, for strong surfactants, the droplet’s surface-area-to-volume ratio becomes the key factor in determining droplet surface tension rather than differences in surfactant properties. For the same total surfactant concentration, the surface tension of a droplet can be >40 mN/m higher than that of the macroscopic solution that produced it. These observations indicate that an explicit consideration of surface-area-to-volume ratios is required when investigating heterogeneous chemical reactivity at the surface of aerosol droplets or estimating aerosol activation to cloud droplets.

## Abstract 2

Surface-area-to-volume ratio becomes the key determinant of surface tension for small droplets containing strong surfactants.

## Introduction

Aerosols, although too small to see, impact many aspects of our lives including climate,^1^ human health,^2^ disease transmission,^3^ and materials synthesis.^4^ Moreover, aerosol droplets are microcompartments where chemical reactions can be accelerated by up to 10^6^ times relative to the rates in macroscopic solutions.^5,6^ In aerosols, interfacial reactivity can dominate bulk reactivity owing to the extremely large surface-area-to-volume (SA-V) ratios accessible in finite-volume droplets.^7^ Surfactants are widely used in a variety of aerosol fields including to tune the morphology of spray-dried particles^8,9^ and to aid in jet breakup in electrospray ionization.^10^ Furthermore, surfactants are commonly found in ambient atmospheric aerosol.^11,12^ Owing to their surface-active nature, these surfactants can undergo unique interfacial reactions distinct from those in the gas phase or in bulk solutions.^13−16^

Surfactants can also reduce the surface tension of aerosol droplets. In the atmosphere, a reduction in surface tension could lower the barrier to forming cloud droplets,^17−20^ leading to significant changes in predicted cloud droplet number concentrations^21−23^ and modifying radiative forcing predictions.^19,23^ However, spirited debate exists over the appropriate value for aerosol surface tension due to uncertainties in surface-bulk partitioning in finite-volume (microscopic) systems.^24,25^ Although surfactants reduce surface tension by adsorbing to the droplet–air interface, such adsorption also depletes the bulk concentration (referred to as bulk depletion), potentially increasing water activity and negating the surface tension lowering impacts of surfactants.^26^ Climate models generally assume that aerosol droplets have a surface tension of pure water throughout the activation process.^27^ However, this assumption is in direct opposition to the routine observation of strong surfactants (i.e., surfactants that can lower the surface tension of macroscopic solutions at concentrations in the mM range and lower) in atmospheric aerosol^11,12,28−30^ and empirical inferences of reduced surface tension to obtain closure (i.e., quantitative agreement) with ambient cloud droplet measurements.^31−33^

A comprehensive understanding of surfactant partitioning in microscopic droplets based on experimental observations has been elusive. This partitioning means that macroscopic surface tension measurements are not directly applicable to picoliter (∼6 μm radius) or smaller volume droplets.^21,34^ A partitioning model, which accounts for the large surface area of the droplet, must be employed,^24,35,36^ but a dearth of experimental surface tension measurements in high SA-V systems impedes the rigorous testing of such models. Few experimental approaches are capable of directly measuring the surface tensions of microscopic droplets,^37−39^ and even fewer have explored the surface tension lowering effects of surfactants in such microscopic systems.^21,40^ In our study, the surface tensions of individual picoliter volume droplets containing surfactants spanning a wide range in critical micelle concentration (CMC), surface activity, or surfactant strength (both defined as inversely proportional to CMC) and molecular structure are investigated. These unique measurements are reconciled with a fully predictive mixed monolayer partitioning model (MLMix) constrained only by independent macroscopic solution measurements. The results provide important insights into how surfactants partition to the droplet–air interface in high SA-V ratio systems, validating thermodynamic approaches to estimate surfactant partitioning and enabling a more nuanced understanding of the capacity for surfactants to lower the surface tension of aerosols, affect cloud formation, and contribute to chemical reactivity.

## Results

The surface tensions of aqueous 6–9 μm radius droplets containing a known concentration of one of six nonionic surfactants—Tween20, octyl-β-d-1-thioglucopyranodide (OTG), or linear poly(oxyethylene) alkyl ethers (C_m_E_n_: C16E8, C14E6, C12E5, and C10E8), see Figure S1—which act as proxies for the nonionic surfactants found in ambient aerosol,^11,12^ and a co-solute (either 0.5 M NaCl, a proxy for sea spray, or 0.9 M glutaric acid, a proxy for water-soluble organic matter) were measured using holographic optical tweezers. (See Methods and SI Methods for full experimental details.) Co-solutes are required in order to lower the droplet water activity and stably trap it in the optical tweezers. Both co-solute concentrations correspond to a water activity of 0.99. The nonionic surfactant–co-solute systems studied here exhibit a wide range of surface activities, with CMCs spanning 0.003–21 mM (Table S1).

**Figure 1:**
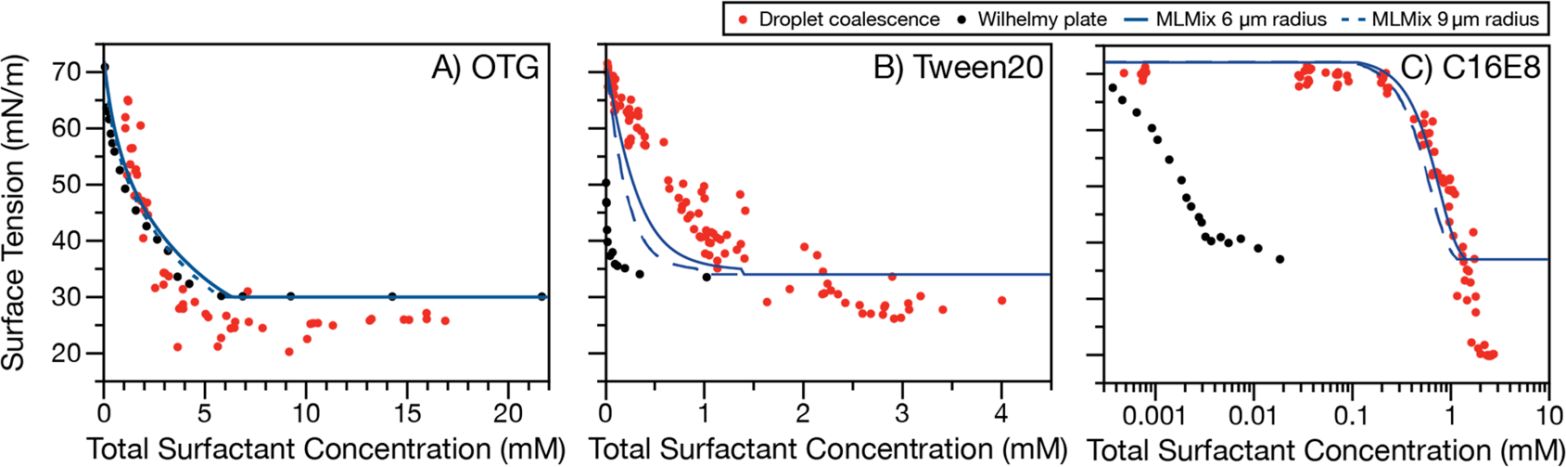
Surface tension of (**A**) OTG, (**B**) Tween20, and (**C**) C16E8 with 0.5 M NaCl co-solute. Macroscopic points (black) are averages of three measurements; error bars representing the standard deviation are smaller than the data marker. Droplet data points (red) each represent a single droplet measurement; an uncertainty on the surface tension can be estimated from the spread in the data points. MLMix predictions for 6 μm (solid blue lines) and 9 μm (dashed blue lines) radius droplets are overlaid. Note the difference in the *x*-axis scales.

Figure 1 compares the surface tensions of picoliter droplets with macroscopic solutions for three surfactants of varying strength (0.5 M NaCl co-solute with OTG, CMC = 6.4 mM; Tween20, CMC = 0.031 mM; and C16E8, CMC = 0.0033 mM) plotted as a function of the total surfactant concentration ([surfactant]_tot_, eq 1), which describes the number of surfactant molecules present both in the bulk of the droplet (*N*_*bulk*_) and at the surface (*N*_*surface*_)
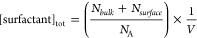
1where *N*_A_ is Avogadro’s number and *V* is the droplet volume.

For the weakest surfactant studied here, there is close agreement between picoliter droplet and macroscopic measurements (Figure 1A). However, for Tween20 (a stronger surfactant; Figure 1B), droplets require a larger total surfactant concentration to reach the minimum surface tension than in macroscopic solutions. For C16E8 (the strongest surfactant studied here, Figure 1C), more than 2 orders of magnitude more surfactant is required to reach the minimum surface tension in droplets compared to macroscopic solutions. Since the bulk concentration of a given surfactant required to reach the minimum surface tension (i.e., the CMC) is the same in a macroscopic solution and a droplet, the differences in the required total surfactant concentrations across the three systems in Figure 1 represent significant differences in the contribution from the number of molecules at the surface to the total surfactant concentration (eq 1). As the surface activity increases in Figure 1 from panel A to panel C, the contribution of molecules at the surface to the total concentration also increases. This direct experimental observation is consistent with surface partitioning and bulk depletion being more significant in finite-volume droplets for stronger surfactants than for weaker ones.^35^

**Figure 2:**
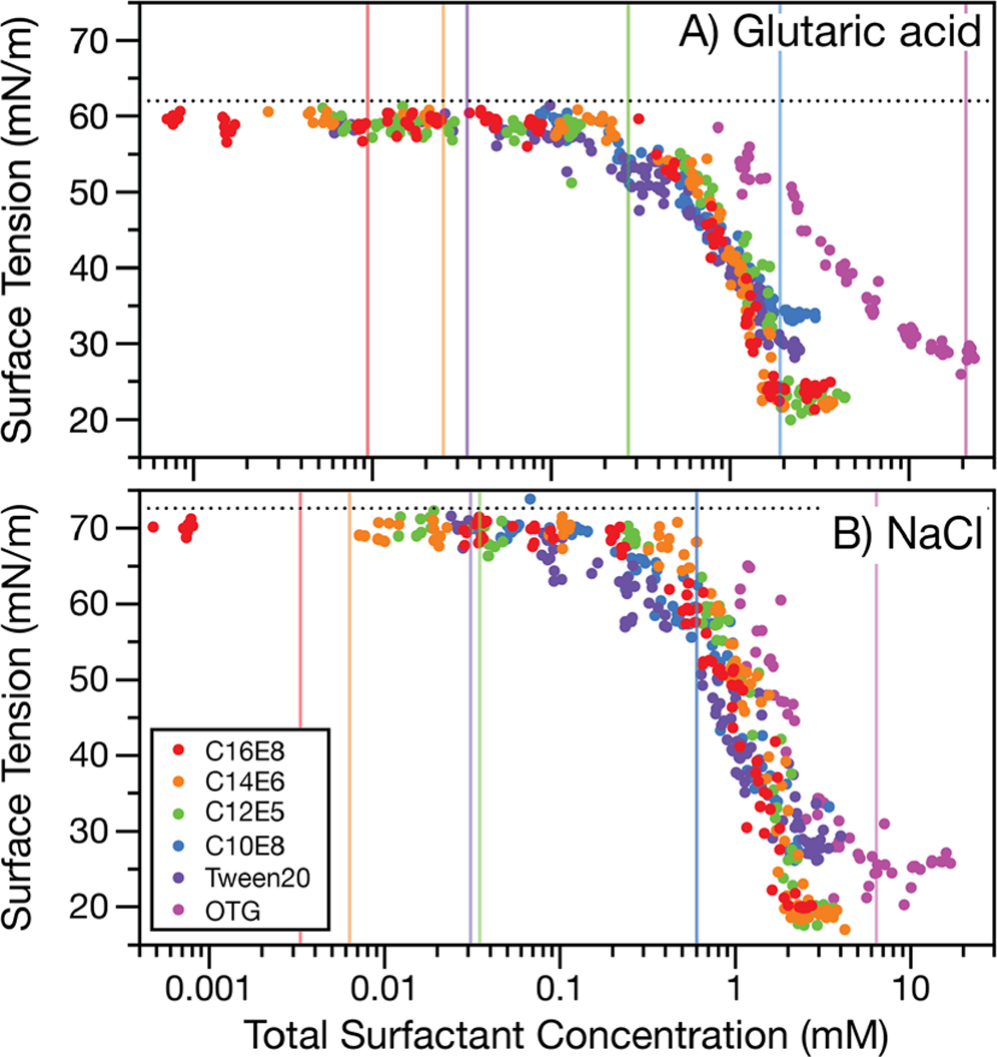
Surface tensions of 6–9 μm radii droplets containing one of six nonionic surfactants and (**A**) 0.9 M glutaric acid or (**B**) 0.5 M NaCl. Vertical lines in the same color as the data points indicate the CMC determined from fitting the Gibbs isotherm to macroscopic surface tension measurements. Horizontal black dotted lines show the expected surface tension for the co-solute solution without surfactant. The uncertainty in each measurement is smaller than that on the data marker.

Picoliter droplet measurements for all studied surfactant–co-solute systems are shown in Figure 2A (0.9 M glutaric acid as a co-solute) and Figure 2B (0.5 M NaCl as co-solute). Each data point represents one measurement of a 6–9 μm radius composite droplet, and each surfactant/co-solute data set consists of ∼90 individual measurements across a wide surfactant concentration range. Vertical lines in the same color indicate the CMC retrieved from the Gibbs isotherm analysis of the macroscopic solutions (Table S1).

Two key observations are evident from Figure 2. First, as the surfactant becomes stronger (i.e., lower CMC), a larger difference between the total surfactant concentration required to reach the minimum surface tension in microscopic droplets relative to macroscopic solutions (indicated by the corresponding vertical line) is observed. This result, consistent with the observation in Figure 1, is found for both NaCl and glutaric acid co-solutes and for surfactants spanning a wide range in surface activities (CMCs spanning <10 μM to ∼1 mM) and chemical structures. The second observation is that all surfactants with greater surface activities than OTG (i.e., all surfactants where bulk depletion is clearly observed) exhibit overlapping trends in surface tension when plotted against the total surfactant concentration.

The overlap of droplet measurements in Figure 2 is explained by the molecular footprints of the surfactant molecules (i.e., the 2D area a surfactant molecule occupies at the interface), which span only a factor of 5 (22–101 Å^2^, see Tables S1 and S2) and are consistent with values reported in the literature.^41,42^ The relatively small range of molecular footprints implies that approximately the same surface concentration is required before the interface becomes saturated and the surface tension no longer decreases with increased surfactant concentration. Only for the least effective surfactant, OTG, which requires a bulk surfactant concentration >6 mM in each mixture to reach the minimum surface tension, is a separate trend observed. Control experiments (see SI text S1) demonstrate that experimental observations cannot be explained by the droplet generation mechanism or surfactant diffusion time scales in droplets. Additionally, we previously showed that the mole ratio of surfactant to co-solute in solution is conserved upon nebulization.^21^

The measured droplet surface tensions can be reconciled with macroscopic solution measurements (i.e., Wilhelmy plate measurements) using a monolayer partitioning model (MLMix) that relies only on macroscopic measurements for each surfactant system (SI Methods). Figure 1 shows predictions of surface tension as a function of total surfactant concentration for 6 and 9 μm radius droplets containing OTG, Tween20, and C16E8 with NaCl co-solute, and Figures S2 and S3 compare MLMix predictions to experimentally measured droplet surface tensions for each of the 12 ternary surfactant–co-solute systems investigated. In Figure 1 and Figure S2 (0.5 M NaCl as co-solute), model-measurement agreement is largely very good. The model predictions exhibit a trend similar to that of the experimental data. Although the model in some cases slightly under- or overpredicts the measured picoliter droplet surface tension, there is no systematic bias in its predictions in the region where surface tension decreases with increased surfactant concentration. In Figure S3 (0.9 M glutaric acid as co-solute), good agreement between the MLMix predictions and experimental measurements is again observed. In the region before the CMC is reached in the droplet bulk, discrepancies between the experimental data and model predictions likely arise from the pseudoternary parametrizations used to describe the systems. Particularly in the case of the NaCl co-solute, it is possible that these parametrizations do not fully capture any potential salting out. Future work will investigate the impact of full ternary parametrizations on model predictions for the surface tension of droplets. Near the CMC, small kinks (e.g., Figure S2E) arise from the model constraint that at the CMC the surface tension of the droplet is equal to the minimum surface tension for the system determined from macroscopic measurements (see SI Methods for further details). The MLMix predictions for droplet surface tension are in much better agreement with the droplet measurement than the macroscopic measurements are with the droplet measurements. If the macroscopic surface tension measurements were taken to be the true surface tension value for the GA-C16E8 system in Figure S3A, the root-mean-square error (RMSE) would be nearly 26 mN/m, more than three times higher than the RMSE calculated from MLMix predictions (see SI text S3 and Table S3). The main discrepancy between droplet measurements and model predictions is in the concentration region where the droplets have reached their minimum surface tension. This discrepancy arises from an enrichment in the surfactant concentration at the droplet interface immediately after coalescence events^21^ and its origin is discussed further in SI Text S2. The RMSE for measurement-model agreement (see SI Text S3) for each ternary system investigated is provided in Table S3.

**Figure 3:**
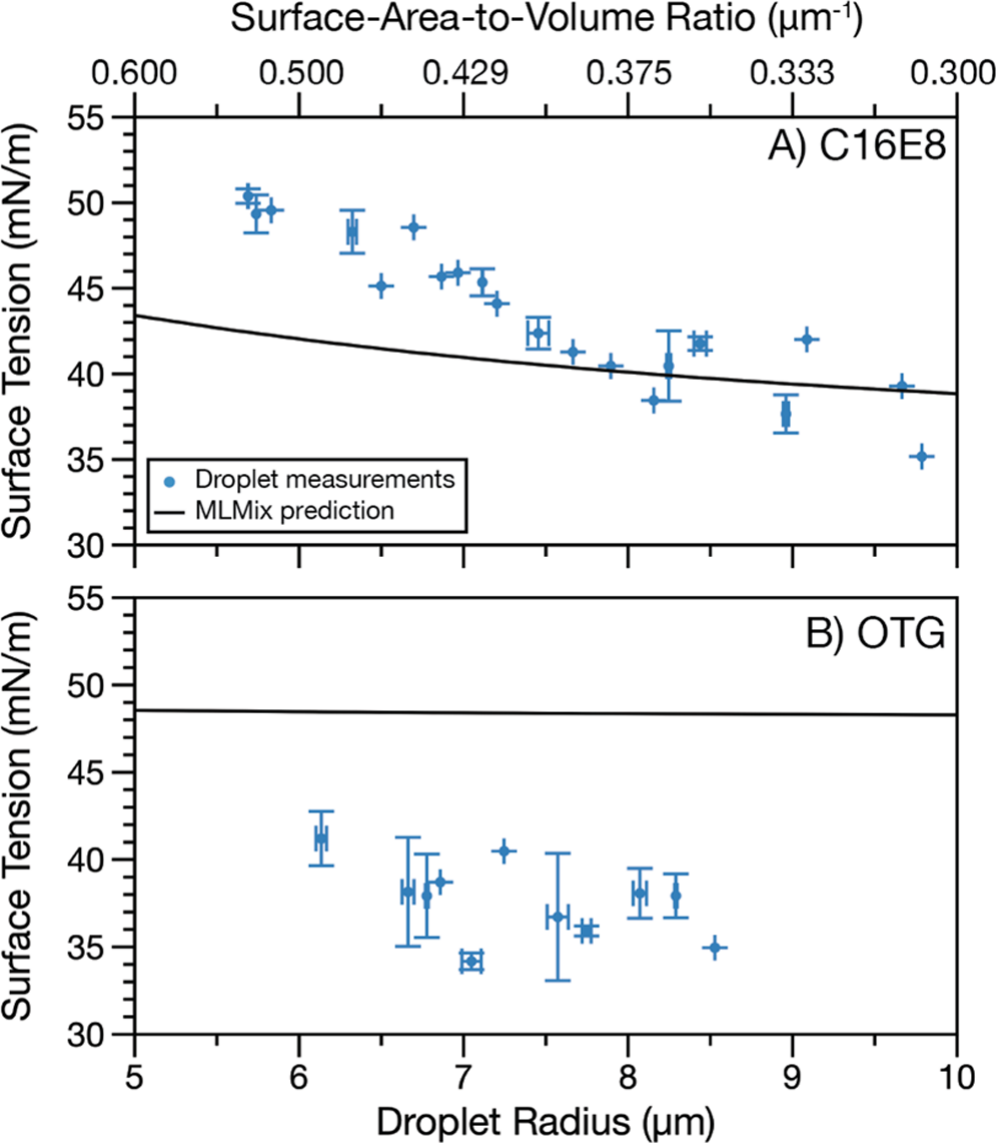
Surface tension as a function of the composite droplet radius for (**A**) 0.8 mM C16E8 in 0.9 M glutaric acid and (**B**) 4 mM OTG in 0.9 M glutaric acid. Data points represent measurements grouped into 0.1 μm bins, and error bars show the standard deviation in concentration and surface tension from two or more measurements. Solid black lines show the MLMix predictions for the surface tension as a function of droplet radius for each system.

A direct consequence of bulk depletion is that at a fixed total surfactant concentration, changes to the droplet SA-V ratio should alter the droplet’s surface composition. Figure 3 explores how droplet size (i.e., the SA-V ratio) alters the droplet surface tension when the total surfactant concentration is fixed. In mixtures with the 0.9 M glutaric acid co-solute, two surfactants are investigated: C16E8 (the strongest surfactant studied, with clear evidence of bulk depletion in Figure 2A) and OTG (the weakest surfactant studied, with no evidence of bulk depletion observed in Figure 2A). For each system, surfactant concentrations were chosen in a region where the surface tension changes steeply with concentration. Measured surface tension as a function of droplet size (5–10 μm radius) is compared to predictions from MLMix. This larger span in radius was selected to better show the size-dependent surface tension behavior. For 0.8 mM C16E8 with glutaric acid co-solute (Figure 3A), a clear size-dependent surface tension is observed in the experimental measurements, with surface tension falling from about 50 mN/m at 5.5 μm droplet radius to about 35 mN/m at 10 μm droplet radius. The model also predicts a size-dependent surface tension, although the dependence is not as strong as observed in the droplet measurements. Nonetheless, there is reasonable agreement between the experimental data and the model predictions. Figure 3B shows size-dependent surface tension measurements for droplets containing 4 mM OTG (glutaric acid co-solute). Although the model overpredicts surface tension by ∼10 mN/m, qualitatively both the measurements and model predictions show very little change in surface tension with droplet radius. The discrepancy between measurements and model predictions could be due to the enrichment of surfactant at the composite droplet interface (see SI text S2.) Nevertheless, this observation is consistent with Figure 2A: no bulk depletion was observed; therefore, altering the SA-V ratio in this size range would not alter the droplet surface composition relative to the corresponding macroscopic solution. For this system, bulk depletion is still expected to occur at higher SA-V ratios.

**Figure 4:**
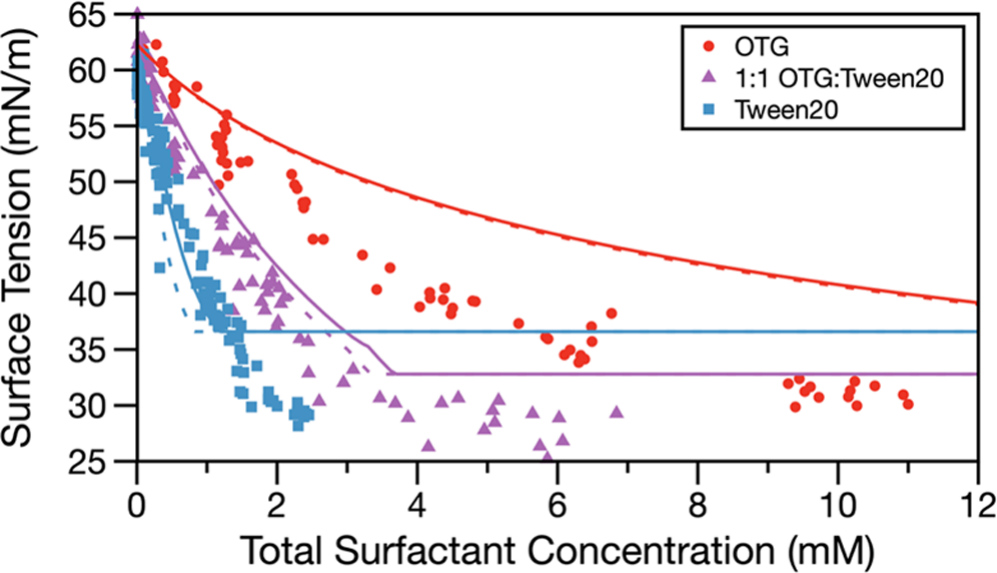
Surface tension measurements and monolayer partitioning model predictions for 6 μm (solid line) and 9 μm (dashed line) radius droplets. Red circles: OTG. Blue squares: Tween20. Purple triangles: 1:1 mol mixture of OTG:Tween20. All droplets contain the 0.9 M glutaric acid co-solute.

The chemical complexity of the droplets was further extended to a mixture containing two strong surfactants, OTG and Tween20, providing the first droplet surface tension measurements where competitive surfactant adsorption is important. Figure 4 shows surface tension measurements of droplets containing a 1:1 mol/mol mixture of OTG:Tween20 and droplets containing only one of these surfactants (all with 0.9 M glutaric acid co-solute). Measured surface tensions for the 1:1 mixture lie in between the values for the two individual surfactants but closer to values for Tween20, which is the more effective surfactant in this mixture. MLMix predictions for the mixed surfactant case (SI Text S3) agree well with the experimental measurements, which is remarkable given the chemical complexity of the system. The RMSE values for model–experiment agreement are in Table S3. This experiment demonstrates the ability of the MLMix model to predict surface tensions of complex chemical mixtures, where multiple surfactants are competing for surface sites.

**Figure 5:**
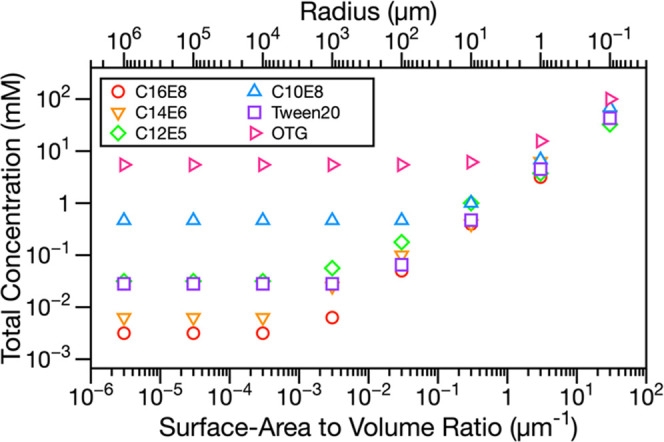
MLMix predictions for the total surfactant concentration required to reach a full monolayer surface coverage (and minimum surface tension) for 0.5 M NaCl and surfactant mixtures in 0.1–10^6^ μm radius droplets. The total concentration is defined in eq 1.

Experimental measurements are limited to droplets ≳5 μm radius (determined by the smallest droplet sizes that can be characterized in the optical trap). However, aerosol droplet sizes often extend to much smaller values, including <100 nm radius. To explore the implications of our experimental observations of bulk depletion, we used MLMix to predict the total surfactant concentration required to reach maximum surface coverage (and minimum surface tension) across 8 orders of magnitude in the droplet SA-V ratio. Figure 5 shows these predictions with 0.5 M NaCl as the co-solute. When the droplet SA-V ratio is small (i.e., the radius is large), the amount of surfactant required to reach a monolayer surface coverage is highly dependent on surfactant properties, with less effective surfactants requiring orders of magnitude more surfactant to reach monolayer coverage than their highly surface active counterparts. These concentration differences are due to the total concentration being dominated by molecules in the bulk. As the SA-V ratio increases (i.e., radius decreases), bulk depletion becomes significant and the total surfactant concentration required to reach monolayer surface coverage increases. This bulk depletion is predicted to become apparent in larger droplets for more effective surfactants than for less effective ones (e.g., in Figure 5, depletion is first observed at *r* = 10^3^ μm for C12E5 but at *r* = 1 μm for OTG). At the largest SA-V ratios, the amount of surfactant required to reach a monolayer surface coverage becomes a much weaker function of the surfactant surface activity than at smaller SA-V ratios. This observation is a consequence of the number of molecules at the surface, constituting a significant fraction of the total concentration. In Figure 5, at a droplet radius of 0.1 μm, all surfactant systems require the same order of magnitude surfactant concentration (between 32 and 91 mM) to achieve full monolayer surface coverage and reduce the surface tension to its minimum value, despite these surfactants requiring orders of magnitude different concentrations to reach the minimum surface tension in macroscopic solutions. Similar trends are observed for surfactant mixtures with 0.9 M glutaric acid (Figure S4). As the SA-V ratio changes, the ratio of the number of surfactant molecules at the droplet interface to that in the droplet bulk also changes. Figure S5 shows this ratio as a function of the SA-V ratio and droplet radius for both 0.5 M NaCl and 0.9 M glutaric acid co-solutes. Using a mole fraction weighted average density for a solid particle with composition shown in Figure 5 and Figure S4, we calculate the corresponding dry particle diameters to be 4.8–5.0 μm, 502–518 nm, and 58–64 nm for 10, 1, and 0.1 μm wet radius droplets containing 0.5 M NaCl, respectively, and 5.5–6.0 μm, 564–600 nm, and 63–71 nm for 10, 1, and 0.1 μm wet radius droplets containing 0.9 M glutaric acid, respectively.

## Discussion

The combined experimental and model results have important implications in many contexts where aerosols play key roles. For example, in atmospheric aerosol, a reduced surface tension owing to surfactants has been inferred to explain significant observed increases in cloud droplet concentrations relative to predictions assuming a pure water surface tension, altering estimations of cloud properties.^31−33^ The surfactants studied in this work cover the broad range of surface activities possessed by atmospheric surfactants.^11,12,30,43^ Our results demonstrate that for sufficiently strong surfactants the concentration required to reduce the surface tension of finite-volume droplets becomes less dependent on a surfactant’s properties. Instead, because many surfactant molecules have similar molecular footprints, the total concentration required to populate the interface is similar. Although ambient aerosols may contain surfactants with very different chemical structures^44,45^ or, in the case of fulvic and humic acids, poorly defined structures,^9,42,45−47^ the surface excess concentrations and molecular footprints reported for these surface-active materials are generally ∼3 × 10^–6^ mol/m^2^ and ∼50 Å^2^, respectively,^9,42,47^ consistent with values for the surfactants studied here. The implication of this result is that when considering the depletion of surfactants in small droplets (e.g., accumulation and ultrafine aerosol modes), the chemical complexity of strong atmospheric surfactants may be reduced to a simple model surfactant with a representative molecular footprint to estimate partitioning behavior. Such a simplification may facilitate explorations of the sensitivity of cloud droplet activation to aerosol surface tension. Indeed, the MLMix predictions in this work suggest that for droplets with a wet radius of 0.1 μm (water activity = 0.99), a total surfactant concentration of <100 mM is required to reach full monolayer coverage. These concentrations are well within the range of concentrations reported for ambient aerosol.^11,12^

Chemical reactions may also proceed at highly accelerated rates (enhancement factors sometimes up to 10^6^) in microcompartments such as aerosol droplets.^48,49^ The aerosol–air interface is central to explanations of reaction acceleration, which include the physical confinement of reagents at the interface in high SA-V droplets, partial solvation at the interface, and interfacial electric fields.^49,50^ Reactions in microdroplets can be both accelerated and inhibited by the presence of surfactants.^5,7^ However, little attention has been paid to surface composition and whether the studied droplets were truly coated with surfactant monolayers. Our study demonstrates that droplet surface composition does not necessarily match that of the macroscopic solution from which the droplet was generated and that droplet surface composition can vary significantly with droplet size solely due to changes in the SA-V ratio. These observations have many implications in terms of size-dependent chemical reactivity, given the significance of interfacial chemistry to aerosols and droplets, and suggest that droplet surface coverage must be explicitly considered in the interpretation of reactions in surfactant-containing droplets.

## Conclusions

In short, we demonstrate for a range of surfactants that finite-volume droplets require significantly higher surfactant loadings in comparison to the corresponding macroscopic solutions to reduce droplet surface tension. These experimental observations are explained fundamentally by considering the very high SA-V ratios inherent in aerosol droplets. The results directly inform key challenges in our understanding of clouds, climate, and chemical reactivity in microcompartments. For strong surfactants, the droplet surface composition is a strong function of droplet size and could result in vastly different chemical reactivity in droplets with the same total composition but different SA-V ratios. Furthermore, the total concentration of surfactant required to reduce the surface tension at 0.99 water activity for 100 nm radius droplets is consistent with levels of surface-active material quantified in ambient samples.^11,12^ These results suggests that under some circumstances, there may be enough surface-active material in aerosol to lower the surface tension near the point of cloud droplet activation. The incorporation of more detailed and accurate representations of aerosol surface tension in cloud parcel models, using parametrizations developed from bulk-to-surface partitioning modeling, is required to quantify the sensitivity of surface tension effects on cloud droplet formation.^22,51^ Finally, future efforts are required to extend the experimental characterization of droplet surface tensions to the submicrometer size range, where depletion is also expected to become important for less surface-active but atmospherically abundant organic molecules.

## Methods

The surface tension of aerosol droplets was measured by coalescing droplets in holographic optical tweezers (see Figure S6).^52^ Droplets contained a co-solute (0.5 M NaCl or 0.9 M glutaric acid) to reduce the water activity to 0.99 and allow stable trapping of the droplet in the optical tweezers. Droplets also contained one or two surfactants over a range of concentrations. (See SI Methods for full details on surfactants and procedures.) To measure the surface tension, first, two droplets of the same composition are confined in two optical traps. The traps are slowly moved together until the droplets meet and coalesce. Upon coalescence, the resulting composite droplet begins to oscillate in the trap. We collected the elastic backscattered light as a function of time, which shows an oscillatory pattern in light intensity. A fast Fourier transform of this pattern yields the oscillation frequency of the droplet. Simultaneous to the collection of elastically scattered light, we collected the cavity-enhanced Raman scattering, which, using Mie theory, allows a high-precision determination of the size and refractive index of the droplet. The refractive index is then used with parametrizations that allow quantification of the concentration of the co-solute, with the assumption that the surfactant does not alter the refractive index. The concentration of the surfactant is then quantified using the mole ratio of surfactant to co-solute in the nebulized solution. From the two scattering spectra, we retrieve all of the information required to calculate the droplet surface tension. The surface tensions of macroscopic solutions for the same co-solute/surfactant mixtures were measured using the Wilhelmy plate method. These measurements were parametrized and used as input for the monolayer partitioning modeling. Full details of the experimental procedure and a description of the modeling approach are provided in the SI Methods section.

## Data Availability

All data underlying the figures are available through the University of Bristol data repository, data.bris, at https://doi.org/10.5523/bris.bi3umjin511z28gg91upzvcxx.

## Supporting Information Available

Full details of the experimental, data processing, and modeling procedures; supplementary text describing control experiments, a description of the origin of the discrepancy between the droplet measurements and model predictions at surfactant concentrations larger than the droplet CMC, a description of the evaluation of the data-model agreement, and additional figures and tables necessary to supplement the discussion (PDF)

## References

[ref1] IPCC, 2021: Climate Change 2021: The Physical Climate Basis. Contribution of Working Group I to the Sixth Assessment Report of the Intergovernmental Panel on Climate Change; Masson-Delmotte V. P., et al., Eds.; Cambridge University Press: Cambridge, U.K., 2021.

[ref2] Vehring R. Pharmaceutical Particle Engineering via Spray Drying. Pharm. Res. 2008, 25 (5), 999–1022. 10.1007/s11095-007-9475-1. 18040761 PMC2292490

[ref3] Wang C. C.; Prather K. A.; Sznitman J.; Jimenez J. L.; Lakdawala S. S.; Tufekci Z.; Marr L. C. Airborne Transmission of Respiratory Viruses. Science 2021, 373 (6558), eabd9149 10.1126/science.abd9149. 34446582 PMC8721651

[ref4] Marchand P.; Hassan I. A.; Parkin I. P.; Carmalt C. J. Aerosol-Assisted Delivery of Precursors for Chemical Vapour Deposition: Expanding the Scope of CVD for Materials Fabrication. Dalt. Trans. 2013, 42 (26), 9406–9422. 10.1039/c3dt50607j. 23629474

[ref5] Marsh B. M.; Iyer K.; Cooks R. G. Reaction Acceleration in Electrospray Droplets: Size, Distance, and Surfactant Effects. J. Am. Soc. Mass Spectrom. 2019, 30 (10), 2022–2030. 10.1007/s13361-019-02264-w. 31410654

[ref6] Wilson K. R.; Prophet A. M.; Rovelli G.; Willis M. D.; Rapf R. J.; Jacobs M. I. A Kinetic Description of How Interfaces Accelerate Reactions in Micro-Compartments. Chem. Sci. 2020, 11 (32), 8533–8545. 10.1039/D0SC03189E. 34123113 PMC8163377

[ref7] Bain R. M.; Pulliam C. J.; Thery F.; Cooks R. G. Accelerated Chemical Reactions and Organic Synthesis in Leidenfrost Droplets. Angew. Chemie - Int. Ed. 2016, 55 (35), 10478–10482. 10.1002/anie.201605899. 27465311

[ref8] Chen Z.; Nan Z. Controlling the Polymorph and Morphology of CaCO3 Crystals Using Surfactant Mixtures. J. Colloid Interface Sci. 2011, 358 (2), 416–422. 10.1016/j.jcis.2011.02.062. 21470618

[ref9] Tuckermann R.; Cammenga H. K. The Surface Tension of Aqueous Solutions of Some Atmospheric Water-Soluble Organic Compounds. Atmos. Environ. 2004, 38, 6135–6138. 10.1016/j.atmosenv.2004.08.005.

[ref10] Badu-Tawiah A.; Cooks R. G. Enhanced Ion Signals in Desorption Electrospray Ionization Using Surfactant Spray Solutions. J. Am. Soc. Mass Spectrom. 2010, 21 (8), 1423–1431. 10.1016/j.jasms.2010.04.001. 20483640

[ref11] Gérard V.; Nozière B.; Fine L.; Ferronato C.; Singh D. K.; Frossard A. A.; Cohen R. C.; Asmi E.; Lihavainen H.; Kiveka N.; Aurela M.; Brus D.; Frka S.; Kusan A. C. Concentrations and Adsorption Isotherms for Amphiphilic Surfactants in PM 1 Aerosols from Different Regions of Europe. Environ. Sci. Technol. 2019, 53, 12379–12388. 10.1021/acs.est.9b03386. 31553874

[ref12] Gérard V.; Nozière B.; Baduel C.; Fine L.; Frossard A. A.; Cohen R. C. Anionic, Cationic, and Nonionic Surfactants in Atmospheric Aerosols from the Baltic Coast at Asko, Sweden: Implications for Cloud Droplet Activation. Environ. Sci. Technol. 2016, 50, 2974–2982. 10.1021/acs.est.5b05809. 26895279

[ref13] Rossignol S.; Tinel L.; Bianco A.; Passananti M.; Brigante M.; Donaldson D. J.; George C. Atmospheric Photochemistry at a Fatty Acid–Coated Air-Water Interface. Science 2016, 353 (6300), 699–702. 10.1126/science.aaf3617. 27516601

[ref14] Griffith E. C.; Carpenter B. K.; Shoemaker R. K.; Vaida V. Photochemistry of Aqueous Pyruvic Acid. Proc. Natl. Acad. Sci. U. S. A. 2013, 110 (29), 11714–11719. 10.1073/pnas.1303206110. 23821751 PMC3718102

[ref15] Rapf R. J.; Perkins R. J.; Dooley M. R.; Kroll J. A.; Carpenter B. K.; Vaida V. Environmental Processing of Lipids Driven by Aqueous Photochemistry of α-Keto Acids. ACS Cent. Sci. 2018, 4, 624–630. 10.1021/acscentsci.8b00124. 29806009 PMC5968514

[ref16] Hayeck N.; Mussa I.; Perrier S.; George C. Production of Peroxy Radicals from the Photochemical Reaction of Fatty Acids at the Air-Water Interface. ACS Earth Sp. Chem. 2020, 4 (8), 1247–1253. 10.1021/acsearthspacechem.0c00048.

[ref17] Lin J. J.; Malila J.; Prisle N. L. Cloud Droplet Activation of Organic-Salt Mixtures Predicted from Two Model Treatments of the Droplet Surface. Environ. Sci. Process. Impacts 2018, 20 (11), 1611–1629. 10.1039/C8EM00345A. 30398264 PMC6716451

[ref18] Prisle N. L. A Predictive Thermodynamic Framework of Cloud Droplet Activation for Chemically Unresolved Aerosol Mixtures, Including Surface Tension, Non-Ideality, and Bulk – Surface Partitioning. Atmos. Chem. Phys. 2021, 21, 16387–16411. 10.5194/acp-21-16387-2021.

[ref19] Prisle N. L.; Asmi A.; Topping D.; Partanen A. I.; Romakkaniemi S.; Dal Maso M.; Kulmala M.; Laaksonen A.; Lehtinen K. E. J.; McFiggans G.; Kokkola H. Surfactant Effects in Global Simulations of Cloud Droplet Activation. Geophys. Res. Lett. 2012, 39 (5), L05802 10.1029/2011GL050467.

[ref20] Hansen A. M. K.; Hong J.; Raatikainen T.; Kristensen K.; Ylisirniö A.; Virtanen A.; Petäjä T.; Glasius M.; Prisle N. L. Hygroscopic Properties and Cloud Condensation Nuclei Activation of Limonene-Derived Organosulfates and Their Mixtures with Ammonium Sulfate. Atmos. Chem. Phys. 2015, 15 (24), 14071–14089. 10.5194/acp-15-14071-2015.

[ref21] Bzdek B. R.; Reid J. P.; Malila J.; Prisle N. L. The Surface Tension of Surfactant-Containing, Finite Volume Droplets. Proc. Natl. Acad. Sci. U. S. A. 2020, 117 (15), 8335–8343. 10.1073/pnas.1915660117. 32238561 PMC7165431

[ref22] Malila J.; Prisle N. L. A Monolayer Partitioning Scheme for Droplets of Surfactant Solutions. J. Adv. Model. Earth Syst. 2018, 10 (12), 3233–3251. 10.1029/2018MS001456. 31007837 PMC6472654

[ref23] Lowe S. J.; Partridge D. G.; Davies J. F.; Wilson K. R.; Topping D.; Riipinen I. Key Drivers of Cloud Response to Surface-Active Organics. Nat. Commun. 2019, 10 (1), 5214 10.1038/s41467-019-12982-0. 31740670 PMC6861266

[ref24] Vepsäläinen S.; Calderón S. M.; Prisle N. L. Comparison of Six Approaches to Predicting Droplet Activation of Surface Active Aerosol - Part 2: Strong Surfactants. Atmos. Chem. Phys. Discussions 2023, 10.5194/egusphere-2022-1188.

[ref25] Davies J. F.; Zuend A.; Wilson K. R. Technical Note: The Role of Evolving Surface Tension in the Formation of Cloud Droplets. Atmos. Chem. Phys. 2019, 19 (5), 2933–2946. 10.5194/acp-19-2933-2019.

[ref26] Sorjamaa R.; Svenningsson B.; Raatikainen T.; Henning S.; Bilde M.; Laaksonen A. The Role of Surfactants in Kohler Theory Reconsidered. Atmos. Chem. Phys. 2004, 4 (8), 2107–2117. 10.5194/acp-4-2107-2004.

[ref27] Tao W. K.; Chen J. P.; Li Z.; Wang C.; Zhang C. Impact of Aerosols on Convective Clouds and Precipitation. Rev. Geophys. 2012, 50, RG2001 10.1029/2011RG000369.

[ref28] Kroflič A.; Frka S.; Simmel M.; Wex H.; Grgić I. Size-Resolved Surface-Active Substances of Atmospheric Aerosol: Reconsideration of the Impact on Cloud Droplet Formation. Environ. Sci. Technol. 2018, 52 (16), 9179–9187. 10.1021/acs.est.8b02381. 30048123

[ref29] Facchini M. C.; Mircea M.; Fuzzi S.; Charlson R. J. Cloud Albedo Enhancement by Surface-Active Organic Solutes in Growing Droplets. Nature 1999, 401 (6750), 257–259. 10.1038/45758.

[ref30] Frossard A. A.; Gérard V.; Duplessis P.; Kinsey J. D.; Lu X.; Zhu Y.; Bisgrove J.; Maben J. R.; Long M. S.; Chang R. Y.; Beaupré S. R.; Kieber D. J.; Keene W. C.; Nozière B.; Cohen R. C. Properties of Seawater Surfactants Associated with Primary Marine Aerosol Particles Produced by Bursting Bubbles at a Model Air – Sea Interface. Environ. Sci. Technol. 2019, 53, 9407–9417. 10.1021/acs.est.9b02637. 31329419

[ref31] Ovadnevaite J.; Zuend A.; Laaksonen A.; Sanchez K. J.; Roberts G.; Ceburnis D.; Decesari S.; Rinaldi M.; Hodas N.; Facchini M. C.; Seinfeld J. H.; O’Dowd C. Surface Tension Prevails over Solute Effect in Organic-Influenced Cloud Droplet Activation. Nature 2017, 546 (7660), 637–641. 10.1038/nature22806. 28636594

[ref32] Irwin M.; Good N.; Crosier J.; Choularton T. W.; Mcfiggans G. Reconciliation of Measurements of Hygroscopic Growth and Critical Supersaturation of Aerosol Particles in Central Germany. Atmos. Chem. Phys. 2010, 10, 11737–11752. 10.5194/acp-10-11737-2010.

[ref33] Good N.; Topping D. O.; Allan J. D.; Flynn M.; Fuentes E.; Irwin M.; Williams P. I.; Coe H. Consistency between Parameterisations of Aerosol Hygroscopicity and CCN Activity during the RHaMBLe Discovery Cruise. Atmos. Chem. Phys. 2010, 10, 3189–3203. 10.5194/acp-10-3189-2010.

[ref34] Lin J. J.; Kristensen T. B.; Calderón S. M.; Malila J.; Prisle N. L. Effects of Surface Tension Time-Evolution for CCN Activation of a Complex Organic Surfactant. Environ. Sci. Process. Impacts 2020, 22 (2), 271–284. 10.1039/C9EM00426B. 31912080

[ref35] Alvarez N. J.; Walker M.; Anna S. L. A Criterion to Assess the Impact of Confined Volumes on Surfactant Transport to Liquid – Fluid Interfaces. Soft Matter 2012, 8, 8917–8925. 10.1039/c2sm25447f.

[ref36] Schmedding R.; Zuend A. A Thermodynamic Framework for Bulk – Surface Partitioning in Finite-Volume Mixed Organic – Inorganic Aerosol Particles and Cloud Droplets. Atmos. Chem. Phys. 2023, 23, 7741–7765. 10.5194/acp-23-7741-2023.

[ref37] Lee H. D.; Estillore A. D.; Morris H. S.; Ray K. K.; Alejandro A.; Grassian V. H.; Tivanski A. V. Direct Surface Tension Measurements of Individual Sub-Micrometer Particles Using Atomic Force Microscopy. J. Phys. Chem. A 2017, 121 (43), 8296–8305. 10.1021/acs.jpca.7b04041. 28981283

[ref38] Rafferty A.; Gorkowski K.; Zuend A.; Preston T. C. Optical Deformation of Single Aerosol Particles. Proc. Natl. Acad. Sci. U. S. A. 2019, 116 (40), 19880–19886. 10.1073/pnas.1907687116. 31527232 PMC6778217

[ref39] Miles R. E. H.; Glerum M. W. J.; Boyer H. C.; Walker J. S.; Dutcher C. S.; Bzdek B. R. Surface Tensions of Picoliter Droplets with Sub-Millisecond Surface Age. J. Phys. Chem. A 2019, 123 (13), 3021–3029. 10.1021/acs.jpca.9b00903. 30864798

[ref40] Alvarez N. J.; Walker L. M.; Anna S. L. A Microtensiometer To Probe the Effect of Radius of Curvature on Surfactant Transport to a Spherical Interface. Langmuir 2010, 26 (16), 13310–13319. 10.1021/la101870m. 20695573

[ref41] Zdziennicka A.; Szymczyk K.; Krawczyk J.; Jańczuk B. Activity and Thermodynamic Parameters of Some Surfactants Adsorption at the Water-Air Interface. Fluid Phase Equilib. 2012, 318, 25–33. 10.1016/j.fluid.2012.01.014.

[ref42] Aumann E.; Hildemann L. M.; Tabazadeh A. Measuring and Modeling the Composition and Temperature-Dependence of Surface Tension for Organic Solutions. Atmos. Environ. 2010, 44 (3), 329–337. 10.1016/j.atmosenv.2009.10.033.

[ref43] Petters S. S.; Petters M. D. Surfactant Effect on Cloud Condensation Nuclei for Two-Component Internally Mixed Aerosols. J. Geophys. Res. Atmos. 2016, 121, 1878–1895. 10.1002/2015JD024090.

[ref44] Ekström S.; Nozière B.; Hultberg M.; Alsberg T.; Magnér J.; Nilsson E. D.; Artaxo P. A Possible Role of Ground-Based Microorganisms on Cloud Formation in the Atmosphere. Biogeosciences 2010, 7 (1), 387–394. 10.5194/bg-7-387-2010.

[ref45] Kristensen T. B.; Prisle N. L.; Bilde M. Cloud Droplet Activation of Mixed Model HULIS and NaCl Particles: Experimental Results and κ-Köhler Theory. Atmos. Res. 2014, 137, 167–175. 10.1016/j.atmosres.2013.09.017.

[ref46] Hayase K.; Tsubota H. Sedimentary Humic Acid and Fulvic Acid as Surface Active Substances. Geochim. Cosmochim. Acta 1983, 47, 947–952. 10.1016/0016-7037(83)90160-6.

[ref47] Lee J. Y.; Hildemann L. M. Surface Tension of Solutions Containing Dicarboxylic Acids with Ammonium Sulfate, D-Glucose, or Humic Acid. J. Aerosol Sci. 2013, 64, 94–102. 10.1016/j.jaerosci.2013.06.004.

[ref48] Banerjee S.; Gnanamani E.; Yan X.; Zare R. N. Can All Bulk-Phase Reactions Be Accelerated in Microdroplets?. Analyst 2017, 142 (9), 1399–1402. 10.1039/C6AN02225A. 28332662

[ref49] Rovelli G.; Jacobs M. I.; Willis M. D.; Rapf R. J.; Prophet A. M.; Wilson K. R. A Critical Analysis of Electrospray Techniques for the Determination of Accelerated Rates and Mechanisms of Chemical Reactions in Droplets. Chem. Sci. 2020, 11 (48), 13026–13043. 10.1039/D0SC04611F. 34094487 PMC8163298

[ref50] Lee J. K.; Walker K. L.; Han H. S.; Kang J.; Prinz F. B.; Waymouth R. M.; Nam H. G.; Zare R. N. Spontaneous Generation of Hydrogen Peroxide from Aqueous Microdroplets. Proc. Natl. Acad. Sci. U. S. A. 2019, 116 (39), 19294–19298. 10.1073/pnas.1911883116. 31451646 PMC6765303

[ref51] Vepsäläinen S.; Calderón S. M.; Malila J.; Prisle N. L. Comparison of Six Approaches to Predicting Droplet Activation of Surface Active Aerosol - Part 1: Moderately Surface Active Organics. Atmos. Chem. Phys. 2022, 22 (4), 2669–2687. 10.5194/acp-22-2669-2022.

[ref52] Bzdek B. R.; Power R. M.; Simpson S. H.; Reid J. P.; Royall C. P. Precise, Contactless Measurements of the Surface Tension of Picolitre Aerosol Droplets. Chem. Sci. 2016, 7 (1), 274–285. 10.1039/C5SC03184B. 28758004 PMC5515047

