# Supplementary material for: Surface-Area-to-Volume Ratio Determines Surface Tensions in Microscopic, Surfactant-Containing Droplets
Source: ACS Cent Sci. 2023 Oct 24;9(11):2076–83. doi: 10.1021/acscentsci.3c00998 (PMC10683496; doi:10.1021/acscentsci.3c00998)
Supplement: Supplementary file 1 — oc3c00998_si_001.pdf [file oc3c00998_si_001.pdf]

Supplementary Information for

Surface-Area-to-Volume Ratio Determines Surface Tensions in Microscopic, Surfactant-Containing Droplets

Alison Bain, Kunal Ghosh, Nønne L. Prisle*, and Bryan R. Bzdek*

Corresponding author: b.bzdek@bristol.ac.uk, nonne.prisle@oulu.fi

The PDF file includes:

Methods
Supplementary Text S1 to S3
Tables S1 to S7
Figures S1 to S10
References

Methods

Experimental Methods

The following chemicals were used without further purification: sodium chloride (NaCl, Sigma-Aldrich BioXtra, >99.5% and Sigma-Aldrich ReagentPlus >99%), glutaric acid (Acros Organics, 99% and Sigma-Aldrich 99%), octyl-β-D-1-thioglucopyranodide (OTG, Sigma-Aldrich >98%), Tween20 (VWR BDH chemicals), octaethylene glycol monohexadecyl ether (C16E8, Sigma-Aldrich BioXtra, > 98%), pentaethylene glycol monododecyl ether (C12E5, Sigma-Aldrich BioXtra, >98%), octaethylene glycol monodecyl ether (C10E8, Sigma-Aldrich BioXtra, >98%), and hexaethylene glycol monoteradecyl ether (C14E6, Sigma-Aldrich BioXtra, >99%). Solutions contained one surfactant and one cosolute or two surfactants and one cosolute and were diluted with deionized water. In order to reach the low surfactant concentrations accurately, stock solutions were made at higher concentrations, and the appropriate volume of stock was added to the flask before dilution. Masses were measured with an analytical balance (±0.0001 g) and all solutions were made with high precision volumetric glassware. The concentration of the cosolute was held constant (0.5 M NaCl or 0.9 M glutaric acid) whereas the concentration of surfactant was varied over a wide range. No unexpected or unusually high safety hazards were encountered.

Droplet Measurements

The surface tension of single picoliter droplets was determined by controlled coalescence of two droplets using the holographic optical tweezers shown in Fig. S6A. This method has previously been described in detail.[1,2] Briefly, a plume of aerosol is generated using a medical nebulizer and drawn into the trapping cell. Two droplets are confined in optical traps generated using a 532 nm laser reflected off a spatial light modulator (SLM) and focused with a high numerical aperture objective. By changing the phase pattern (kinoform) on the SLM, the two traps are slowly brought closer together until the two droplets coalesce. The coalescence event leads to oscillations in the droplet surface, resulting in time-dependent oscillations in the elastic back-scattered laser light (EBL). This signal is collected with a photodiode (Fig. S6B). The cavity-enhanced Raman spectrum of the composite droplet is collected simultaneously and the positions of the resonances are fit with Mie theory to determine the radius and refractive index dispersion of the resulting droplet (Fig. S6C).[3,4]

Droplet surface tension (σ, Eq. 2) is related to the oscillation frequency (ω_l) of the surface mode order l, which can be found by taking a fast-Fourier transform of the EBL signal (shown in Fig. S6B), as well as the droplet radius (r) and density (ρ).

$$\sigma = \frac{r^3 \rho \omega_l{}^2}{l(l-1)(l+2)} \tag{2}$$

The observed oscillation frequency (ω_l^*) was corrected for damping due to the viscosity (η) of the droplet assuming the viscosity of the droplet containing surfactant was equal to that of a binary water-cosolute droplet using Eqs. 3 & 4.[5,6]

$$\omega_l^* = \sqrt{\omega_l^2 - \tau_l^{-2}} \tag{3}$$

$$\tau_l = \frac{r^2 \rho}{(l-1)(2l+1)\eta} \tag{4}$$

Droplet radii were obtained by fitting the cavity-enhanced Raman spectrum of the composite droplet after coalescence. The refractive index at 589 nm (n(589nm)), calculated from the refractive index dispersion obtained from Mie theory fitting, was used to determine the concentration of the cosolute, assuming the surfactant did not alter the refractive index. The concentration of surfactant was then determined using the molar ratio of surfactant to cosolute in the nebulized solution (Fig. S6C). We previously showed that the mole ratio of surfactant to cosolute in solution is conserved upon nebulization.[2] The droplet composition was also used to determine the droplet density. The assumption that the surfactant does not significantly affect the refractive index, density, or viscosity of the droplet is reasonable since the concentration of surfactant (<25 mM) is very low compared to the concentration of the cosolute (0.5 or 0.9 M) and was confirmed with density and n(589 nm) measurements for solutions containing surfactant concentrations much greater than the critical micelle concentration (CMC). Parameterizations used for determining droplet composition, density, and viscosity[1,6,7] are found in Table S4.

Macroscopic Measurements

The equilibrium surface tension of macroscopic solutions containing surfactant and cosolute were measured with the Wilhelmy plate method (Krüss, K100) at a temperature of 25±1°C. Reported surface tensions are an average of three repeat measurements. Agreement between measurements of each solution was within 1.5 mN/m. Surface tension as a function of surfactant concentration in the linear region (i.e. the region where increasing surfactant concentration is proportional to a decrease in surface tension) was fit with the Gibbs isotherm to determine the maximum surface excess of surfactant,

$$\Gamma_{\max} = -\frac{1}{nRT}\left(\frac{\partial\sigma}{\partial \ln(c/c_0)}\right)_T \tag{5}$$

where σ is the surface tension, c is the surfactant concentration normalized by unit concentration c_0, n is the van't Hoff factor for the surfactant at the surface (set to 1 here for nonionic surfactants), R is the gas constant and T is the temperature.

The macroscopic surface tension data were also fit with the Langmuir isotherm (Eq. 6) using the Frumkin equation of state (Eq. 7). Here, Γ is the surface coverage and is a function of the total surfactant concentration, $a = \frac{\beta}{\alpha}$ is the ratio of surface adsorption (β) to desorption (α) rate constants (with units m^3/mol) and Π is the surface pressure, which is defined as the difference between the solvent surface tension (σ_0) and the equilibrium surface tension at a given surface excess (σ).

$$\Gamma = \Gamma_{\max}\left(\frac{ac}{1+ac}\right) \tag{6}$$

$$\Pi = \sigma_0 - \sigma = -nRT\ \Gamma_{\max}\ln\left(1 - \frac{\Gamma}{\Gamma_{\max}}\right) \tag{7}$$

The molecular footprint (i.e. the 2D area a surfactant molecule takes up on the surface) can then be estimated from the maximum surface excess ($\Gamma_{\max}$) and Avogadro's number (N_A),

$$\text{Footprint} = \frac{1}{N_A \Gamma_{\max}}. \quad (8)$$

At surfactant concentrations above the CMC, the change in surface tension with increasing surfactant concentration approaches zero and the minimum surface tension is reached. CMCs for surfactant/cosolute mixtures were determined by calculating the point of intersection between the Gibbs isotherm and a linear fit in this surface tension plateau region.

All macroscopic surface tension data were fit with Gibbs and Langmuir isotherms to determine the surfactant parameters CMC, Γ_{max}, a, and molecular footprint at the surface. These parameters are tabulated for each system in Tables S1 and S2. All Γ_{max} and a values are in the range typically reported for surfactant systems. Note that Γ_{max} is typically on the order of 3×10^{-6} mol/m^2 for surfactants with linear alkyl chain tail but a can vary by orders of magnitude.[8] In general, we find that Γ_{max} is larger for a given surfactant in a solution with 0.5 M NaCl than with 0.9 M glutaric acid. This means that when mixed with 0.9 M glutaric acid, the maximum number of molecules that adsorb to the surface is smaller than in 0.5 M NaCl. A direct consequence of this difference in surface excess is that the estimated average molecular footprint at the surface is higher for mixtures of surfactants with 0.9 M glutaric acid, indicating that the surfactant molecules do not pack as tightly at the surface when glutaric acid is present as when NaCl is present. This observation is likely due to the modest surface propensity of glutaric acid, leading to some competition for surface sites between the surfactant and glutaric acid molecules.

We also see, in general, that the CMCs for surfactants in mixtures with 0.9 M glutaric acid are higher than for 0.5 M NaCl. Measured CMCs for these six surfactants in binary aqueous solution without the presence of any cosolute agree well with literature data[9–18] and fall in between the CMCs found for mixtures with glutaric acid and NaCl (Fig. S7 and Table S5) indicating that the presence of glutaric acid increases the solubility of these surfactants whereas NaCl induces salting out of surfactants. Tween20 is an exception to this trend, and the CMC in the presence of NaCl or glutaric acid cosolutes did not differ significantly from that for the binary aqueous solution.

Surface Partitioning in Droplets

The surface tension of aqueous droplets is calculated based on the composition of the bulk phase, which is determined from the total (superscript t) droplet composition $\mathbf{n}^t = (n_1^t, n_2^t, \ldots)$ (where n is the molar amount) by accounting for size-dependent bulk-to-surface partitioning using the mixed surface monolayer (MLMix) model of Malila and Prisle.[19] A finite-sized spherical droplet with radius r is comprised of a surface monolayer with thickness δ and an interior (bulk) of radius $r - \delta$. The surface is described as a separate liquid phase with a composition distinct from that of the bulk. All species $i = \{\text{water}, \text{surfactant}, \text{cosolute}\}$ in the droplet can partition between these phases.

The compositions of the droplet bulk (superscript b) $\boldsymbol{\chi}^b = (\chi_1^b, \chi_2^b, \ldots)$ and surface (superscript s) $\boldsymbol{\chi}^s = (\chi_1^s, \chi_2^s, \ldots)$ are calculated iteratively using the semi-empirical relation

$$\sigma(\boldsymbol{\chi}^b) = \frac{\sum_i \chi_i^s v_i \sigma_i}{\sum_i \chi_i^s v_i} \quad (9)$$

between the droplet surface tension σ, parameterized in terms of the composition of the bulk (left side Eq. 9), and weighted by the volumes of individual components in the surface (right side Eq. 9). Here, χ_i^b and χ_i^s are the bulk and surface mole fractions, corresponding to molar amounts n_i^b and n_i^s, respectively, v_i are the molecular volumes, and σ_i are pure compound surface tensions of each droplet component i.

Mass conservation, $n_i^t = n_i^s + n_i^b$, is imposed as boundary condition for each component i in the droplet. The mass balance for the surface phase is

$$\sum_i n_i^s m_i = \rho\,(\boldsymbol{\chi}^s) V^s \quad (10)$$

where m_i is the molecular mass of each component i (see Table S6), $\rho(\boldsymbol{\chi}^s)$ is the bulk density of a liquid with the composition of the monolayer, and the volume of the monolayer is determined as $V^s = 4\pi[r^3 - (r - \delta)^3]/3$. The surface monolayer thickness is estimated as the average diameter of molecules in the surface

$$\delta = \left(\frac{6}{\pi}\sum_i v_i \chi_i^s\right)^{\frac{1}{3}}. \quad (11)$$

Major advantages of the monolayer model are that it is fully predictive, such that no parameter fitting to the experimental data being described (droplet measurements) is required, and it explicitly calculates the full composition of both the surface monolayer and bulk phase as functions of the droplet composition and size. The only experimental data used to constrain the model are the independently measured macroscopic solution surface tensions and the solution density (see below). In all estimations of thermodynamic properties, the temperature is assumed constant T = 298 K.

We use a modified Szyszkowski–Langmuir parameterization to fit the measured macroscopic solution surface tensions for ternary and quaternary aqueous solutions. For the ternary water-cosolute-surfactant system, the parameterization has the form

$$\sigma = \sigma_{\mathrm{bin}} - \frac{a_1[surfactant] + a_2[cosolute] + a_3[surfactant][cosolute]}{[surfactant] + [cosolute]} \times \{\ln(1 + b_1[cosolute]) + \ln(1 + b_2[cosolute])\}\,, \quad (12)$$

where σ_{bin} is the surface tension of binary cosolute-water solution obtained from reported literature (ref [20] for water-glutaric acid and ref [21] for water-NaCl), $[surfactant]$ and $[cosolute]$ are their volumetric concentrations, and $a_1, a_2, a_3, b_1,$ and b_2 are the fitting parameters (see Table S7).

For quaternary water-glutaric acid-surfactant1-surfactant2 solutions of several co-adsorbing surfactants, we expand the bulk surface tension parameterization as

$$\sigma = \sigma_{\mathrm{bin}} - \frac{a_1[surfactant1+surfactant2]+a_2[glutaric\ acid]+a_3[surfactant1+surfactant2][glutaric\ acid]}{[surfactant1+surfactant2]+[glutaric\ acid]} \times\{\ln(1+b_1[glutaric\ acid])+\ln(1+b_2[surfactant1+surfactant2])\}, \tag{13}$$

where σ_{bin} is the surface tension of binary aqueous glutaric acid, $[surfactant1 + surfactant2]$ is the total volumetric surfactant concentration, and $a_1, a_2, a_3, b_1,$ and b_2 are the fitting parameters (see Table S7). The standard error of regression (SER) from the least squares fit are calculated for all the ternary and quaternary systems presented in this work (Table S7). SERs are slightly higher for NaCl-surfactant systems compared to the glutaric acid-surfactant systems.

The surface tensions of pure, non-aqueous surfactants required in Eq. 9 are not known and are therefore approximated by the surface tensions at the CMC of each surfactant in a binary aqueous solution, σ_{CMC}. This assumption corresponds to assuming that a complete, pure monolayer with $\chi^s_{surfactant} = 1$ has formed at the CMC and may therefore in some cases lead to discontinuous changes in droplet surface tension σ and χ^s_i when $\chi^b_{surfactant}$ reaches the CMC.

For droplets containing multiple surfactants (here aqueous solutions of a 1:1 molar mixture of Tween20 and OTG with glutaric acid as cosolute), we assume that when the droplet bulk concentration corresponding to the CMC of the surfactant with the lower binary aqueous CMC is reached, a pure monolayer of this surfactant is formed, i.e.

$$\chi^s_{surfactant1} = 1 \ \ if \ \chi^b_{surfactant1,CMC} < \chi^b_{surfactant2,CMC} \tag{14}$$

$$\chi^s_{surfactant2} = 1 \ \ if \ \chi^b_{surfactant2,CMC} < \chi^b_{surfactant1,CMC} \tag{15}$$

Under these conditions, σ_{CMC} is assumed to be equal to the surface tension of the corresponding surfactant (surfactant 1 for Eq. 14 and surfactant 2 for Eq. 15) binary aqueous solution at the CMC. Furthermore, we assume that when $\chi^s_{surfactant1} + \chi^s_{surfactant2} = 1$, and both $\chi^s_{surfactant1}$ and $\chi^s_{surfactant2}$ are non-zero, σ_{CMC} is equal to the surface tension of the 1:1 molar mixture of surfactant 1 (Tween20) and surfactant 2 (OTG) at the average apparent CMC obtained from experimental macroscopic data (See Table S3). The formation of a pure surface monolayer limits further partitioning between the bulk and surface phases in the droplet.

The surface tension and density of pure, sub-cooled NaCl (required for Eqs. 9 and 10) are extrapolated from the correlations presented by Janz et al.[22] For glutaric acid, pure component density and surface tension are taken from the parameterizations presented by Gaman et al.[20] The liquid phase densities $\rho(\boldsymbol{\chi}^s)$ for ternary and quaternary water-surfactant-cosolute bulk solutions are estimated by combining ideal pseudo-binary mixtures of aqueous glutaric acid or NaCl and surfactant using the method of Kodama and Miura[23]:

$$\rho(\boldsymbol{\chi}^s) = \frac{\rho_{ws}\chi^s_{ws}m_{ws}+\rho_{surf}\chi^s_{surf}m_{surf}}{\sum_i m_i\chi^s_i}. \tag{16}$$

Here, ρ_{ws} is the density of the pseudo-binary mixture of water-cosolute, χ^s_{ws} is the total surface mole fraction of water and cosolute $\chi^s_{ws} = \chi^s_{water} + \chi^s_{cosolute})$, m_{ws} is the molecular weight of

the same mixture ($m_{ws} = m_{water}\chi^{s}_{water} + m_{cosolute}\chi^{s}_{cosolute}$), and $\rho_{surf,}$ χ^{s}_{surf} and m_{surf} are the density, surface mole fraction, and molecular weight of the surfactant. ρ_{ws} for mixtures of aqueous glutaric acid-NaCl are obtained from literature[24,25] and the supercooled densities (see Table S6) are used for $\rho_{surf.}$

Supplementary Text

S1: Control Experiments

Two control experiments were carried out to demonstrate that the overlap of all droplet surface tension measurements for strong surfactants in Fig. 2 was not an experimental artefact. First, different droplet generation approaches were explored on the hypothesis that the vibrating mesh nebulizer (OMRON Micro AIR) could alter droplet surfactant concentration by scavenging some surfactant. Figure S8 shows measurements on the glutaric acid/C16E8 system using a nebulizer relying on sonication (OMRON NE-U07) to generate the initial aerosol plume. These data compare favorably with measurements using the vibrating mesh nebulizer across two orders of magnitude in surfactant concentration, suggesting the droplet generation mechanism does not affect the final droplet composition.

The second control experiment explored the possibility of long surfactant diffusional timescales resulting in droplet surface concentrations not reaching equilibrium before coalescence in the optical trap. To test for a diffusion limitation, aerosol droplets were trapped in the two optical traps and held in place for time periods varying from a few seconds to ~30 min before coalescing the two droplets to determine the surface tension. Figure S9 shows that the surface tension lowers by a small amount (5 mN/m over 30 min) with increasing delay times for two concentrations of C16E8 with glutaric acid cosolute. However, this observation is easily explained by dry air from the lab mixing into the trapping cell leading to droplet evaporation. Evaporation increases solute concentrations (evidenced by the increases in droplet refractive index in Fig. S9B). Therefore, as water from the droplets evaporates, the surfactant concentration also increases, reducing droplet surface tension. Note that macroscopic solutions did not require more than 30 min to reach equilibrium surface composition and, due to their small size and surface curvature, mass flux to the surface of droplets is faster than to flat surfaces.[26] Therefore, diffusion limitations do not explain the overlap of droplet surface tension data observed in Fig. 2.

S2: Difference Between Droplet and Macroscopic Solution Minimum Surface Tensions

The difference in minimum surface tension between the macroscopic solution measurements and the droplet measurements, $\Delta\sigma^{min} = \sigma^{min}_{macroscopic} - \sigma^{min}_{droplet}$ is plotted in Fig. S10 against the bulk CMC and demonstrates that $\Delta\sigma^{min}$ is larger for stronger surfactants. This discrepancy in σ^{min} between macroscopic solution measurements and microscopic droplet measurements may arise from the formation of a condensed film upon droplet coalescence,[2] which could lead to a reduced surface tension relative to equilibrium conditions.[27] Upon coalescence of two droplets into one composite droplet, volume is conserved but surface area decreases by a factor of approximately 0.8. To re-establish equilibrium, some surfactant molecules must desorb from the surface and go back into solution. The rate of desorption from the interface will be unique to each surfactant system and will depend on its desorption rate constant, α. The droplet shape oscillations are only detectable for ~100 μs after coalescence before viscous damping returns the droplet to its spherical equilibrium shape, putting a time limit on our surface tension retrieval. Quantitative measurement of α is challenging, but a, the ratio of the adsorption and desorption rate constants, can be

quantified by applying the Langmuir isotherm to the macroscopic solution data. If a is large, adsorption dominates; if a is small, desorption dominates. Table S2 shows that a is large for the more effective C_mE_n surfactants and decreases dramatically for OTG, the least effective surfactant.

The condensed film hypothesis can be used to understand the discrepancies between the model predictions and the experimental data in the region where the total droplet concentration is sufficient to reach the droplet bulk CMC. A condensed film or surface enrichment will decrease the surface tension, and the observed oscillation frequency. If this surface enrichment were present at all surfactant concentrations, we would expect the model to consistently overpredict the surface tension at all concentrations. However, the model agrees with or underpredicts the surface tension at concentrations below the droplet bulk CMC for nearly all studied systems. The only exception is the OTG-glutaric acid system, suggesting surface enrichment may slightly bias measurements at concentrations below the CMC for this system.

S3: Comparison of Monolayer Model Predictions and Experimentally Determined Surface Tensions

The root mean square error (RMSE) is defined as

$$\boldsymbol{RMSE} = \sqrt{\frac{\sum_{i=1}^{n}(\boldsymbol{M_i} - \boldsymbol{E_i})^2}{\boldsymbol{n-1}}} \tag{9}$$

where $\boldsymbol{M_i}$ and $\boldsymbol{E_i}$ are the model prediction and experimentally measured surface tension, respectively, for data point $\boldsymbol{i}$ in $\boldsymbol{n}$ datapoints. For our case where the model predicts two boundaries, droplets having a radius of 6 and 9 μm, we expect all droplet measurements (for droplets in this size range) to fall within these boundaries if the model and experiment were in complete agreement. To calculate the RMSE under these circumstances, the residual, $\boldsymbol{M_i} - \boldsymbol{E_i}$, is taken to be zero if $\boldsymbol{M_i^6} < \boldsymbol{E_i} < \boldsymbol{M_i^9}$, where $\boldsymbol{M_i^6}$ and $\boldsymbol{M_i^9}$ are the model predictions for 6 and 9 μm radii, respectively. If $\boldsymbol{E_i} < \boldsymbol{M_i^6}$ the residual is taken as $\boldsymbol{M_i^6} - \boldsymbol{E_i}$ and if $\boldsymbol{E_i} > \boldsymbol{M_i^9}$ the residual is taken as $\boldsymbol{M_i^9} - \boldsymbol{E_i}$. With this modified definition of RMSE for our prediction envelope defined by two limits in droplet radius, we can determine how well the models agree with the experimental data.

Table S3 shows the RMSE for the experimental surface tension measurements and the monolayer model. The RMSE is calculated for each surfactant in ternary solution with 0.5 M NaCl and 0.9 M glutaric acid (all panels in Figs. S2 & S3) as well as the mixed surfactant system shown in Fig. 4. First, an RMSE was calculated using the entire set of datapoints. The RMSE was then calculated using a reduced dataset which removed measurements with concentrations greater than the apparent CMC as predicted by MLMix. The apparent CMC (the point where droplet measurements are observed to reach a plateau in surface tension) was taken to be the midpoint between the predicted CMCs for droplets of 6 and 9 μm in each case. This reduced dataset removes (at least some of) the experimental points where the experimentally determined surface tension is much lower than the expected equilibrium value, possibly due to the formation of a condensed film (described in Supplementary Text S2). Removing concentrations greater than the CMC tends to reduce the RMSE. All calculated RMSEs fall between 2 and 10 mN/m. If the macroscopic surface

tension measurements are taken to be the true surface tension value for the GA-C16E8 system and 6 – 9 μm droplets, the RMSE would be nearly 26 mN/m. This large increase in error between the droplet measurements and model when the macroscopic surface tension measurements are used without accounting for depletion further illustrates the importance of accounting for depletion in surfactant containing aerosol droplets.

Table S1. Gibbs isotherm parameters from fits of macroscopic surfactant solution measurements.

Surfactant	0.9 M Glutaric Acid			0.5 M NaCl		
	CMC (mM)	Γ_{max} x10^6 (mol/m^2)	Molecular footprint ($Å^2$)	CMC (mM)	Γ_{max} x10^6 (mol/m^2)	Molecular footprint ($Å^2$)
C16E8	0.0094±0.0005	3.1±0.5	54±9	0.0033±0.0003	6±1	27±5
C14E6	0.025±0.002	2.0±0.3	84±14	0.0063±0.0007	3±1	54±22
C12E5	0.27±0.01	2.3±0.1	71±3	0.035±0.002	3.4±0.5	49±7
C10E8	1.9±0.2	1.86±0.08	89±4	0.6±0.1	2.4±0.2	70±6
Tween20	0.034±0.003	3.0±0.6	55±10	0.031±0.003	4.2±0.9	39±8
OTG	20.8±0.4	3.26±0.08	51±1	6.4±0.2	4.1±0.1	41±1

Table S2. Langmuir isotherm parameters from fits of macroscopic surfactant solution measurements.

Surfactant	0.9 M Glutaric Acid			0.5 M NaCl		
	Γ_{max} x10^6 (mol/m^2)	a (m^3/mol)	Molecular footprint (Å^2)	Γ_{max} x10^6 (mol/m^2)	a (m^3/mol)	Molecular footprint (Å^2)
C16E8	4.3±0.3	958±114	38±2	7.6±0.8	1310±271	22±2
C14E6	2.05±0.06	9377±1289	81±3	1.65±0.08	3257±1083	101±5
C12E5	2.32±0.05	774±67	72±2	3.5±0.1	4086±693	48±2
C10E8	1.69±0.07	239±50	98±4	2.2±0.1	1521±371	74±3
Tween20	2.6±0.2	892±173	65±4	4.5±0.3	705±132	37±3
OTG	3.6±0.1	1.6±0.1	46±1	4.7±0.2	7.3±0.8	36±1

Table S3. RMSE for monolayer model prediction and experimentally determined surface tensions as well as average apparent droplet CMC values.

	0.5 M NaCl			0.9 M Glutaric Acid		
Surfactant	Full dataset RMSE (mN/m)	Average apparent CMC (mM)	Concentration < CMC RMSE (mN/m)	Full dataset RMSE (mN/m)	Average apparent CMC (mM)	Concentration < CMC RMSE (mN/m)
C16E8	7.40	1.33	2.88	7.64	1.25	2.89
C14E6	9.97	2.05	7.65	6.22	1.47	2.69
C12E5	8.65	2.22	6.62	5.81	1.70	3.47
C10E8	7.79	1.99	7.25	4.25	2.39	4.12
Tween20	7.54	1.18	6.52	3.24	1.01	2.23
OTG	6.46	6.16	5.86	6.78	26.19	6.78
1:1 mole OTG:Tween20				3.41	3.54	2.45

Table S4. Fitting parameters for polynomial parameterizations (ax^3+bx^2+cx+d) used to determine the cosolute concentration, droplet density, and droplet viscosity.

	x	a	b	c	d
**NaCl**					
Concentration (M) [7]	n(589nm)	-1321.07	5719.68	-8109.36	3775.62
Density (kg/m^3) [1]	Molar concentration	0	$3.506x10^{-3}$	35.06	1000.6
Viscosity (Pa·s) [1]	Molar concentration	0	$4x10^{-5}$	$1x10^{-4}$	$1.1x10^{-3}$
**Glutaric acid**					
Concentration (M) [1]	n(589nm)	5.8647	12.804	2.4342	39.875
Density (kg/m^3) [1]	Molar concentration	0	-0.4163	32.736	997.28
Viscosity (Pa·s) [6]	Molar concentration	0	$9.52x10^{-5}$	$1.509x10^{-4}$	$9.017x10^{-4}$

Table S5. CMCs from the literature (CMC_{lit}) and measured surfactant parameters for aqueous surfactants using the Gibbs isotherm. CMC_{exp} is determined from the intersection of the Gibbs isotherm with a linear fit to the minimum surface tension region.

Surfactant	CMC_{lit} (mM)	CMC_{exp} (mM)	Γ_{max} x10^6 (mol/m^2)	Molecular footrpint (Å^2)
C16E8	0.001-0.016 [9,10,18]	0.0061±0.0006	3.7±0.7	45±8
C14E6	0.0063-0.01 [9,11]	0.0077±0.0008	3.0±0.5	55±10
C12E5	0.064-0.07 [9,12]	0.063±0.004	3.0±0.3	55±5
Tween20	0.06-0.08 [13,14]	0.053±0.004	2.9±0.5	57±11
C10E8	0.97-1.1 [9,15]	1.5±0.4	3.37±0.05	49.3±0.7
OTG	8.5-9.2 [16,17]	9.1±0.3	3.9±0.1	42±1

Table S6. Molecular weight and density for all solution components used in MLMix.

Surfactant	Molar mass (g/mol)	Density (g/cm^3)
C16E8	594.861	0.9987*
C14E6	478.703	0.9984*
C12E5	406.597	0.963†
C10E8	510.702	0.9987*
Tween20	1227.53	1.1†
OTG	308.43	0.9998*
Glutaric acid	132.115	1.4149 [20]
NaCl	58.4428	1.9772 [22]
Water	18.0153	0.9983 [28]

*Measured here for binary aqueous-surfactant solutions where surfactant concentration >> CMC. † Stated on bottle from chemical supplier.

Table S7. Fitting parameters and standard error of regression (SER) for the Szyszkowski–Langmuir parameterization of the macroscopic bulk solution surface tension data.

Surfactant	a_1 (N/m)	a_2 (N/m)	a_3 (N/mM·m)	b_1 (mM^{-1})	b_2 (mM^{-1})	SER (N/m)
**0.9 M Glutaric Acid**						
C16E8	0.087343	0.0061122	-0.100950	-0.2715400	3470.10	0.000798
C14E6	0.108710	0.0045747	-0.101430	0.0060938	9231.10	0.000474
C12E5	0.103640	0.0054407	-0.107070	0.0116870	659.32	0.000413
C10E8	0.102040	0.0034107	-0.108840	0.0035685	313.99	0.000691
Tween20	0.050254	0.0064715	-0.055274	-0.0798380	748.83	0.001310
OTG	0.033005	0.0114380	-0.074442	0.0376540	773.48	0.000468
OTG+Tween20	0.054072	0.0029460	-0.051031	-0.0651870	5128.80	0.001440
**0.5 M NaCl**						
C16E8	0.251000	0.0072440	-0.48230	-0.044224	1700.60	0.00228
C14E6	0.250000	0.0058200	-0.49900	-0.037000	20856.00	0.00249
C12E5	0.251140	0.0085308	-0.49773	-0.063328	3771.50	0.00138
C10E8	0.027461	0.0080942	-0.04508	0.073147	332.18	0.00050
Tween20	1.239300	0.0162900	-2.52140	0.123440	331.96	0.00190
OTG	1.294400	0.0107920	-2.41130	-0.099817	6872.10	0.00110

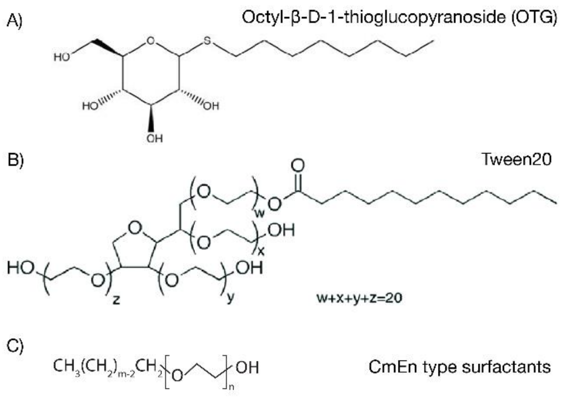

Figure S1: Chemical structures of surfactants used in this investigation.

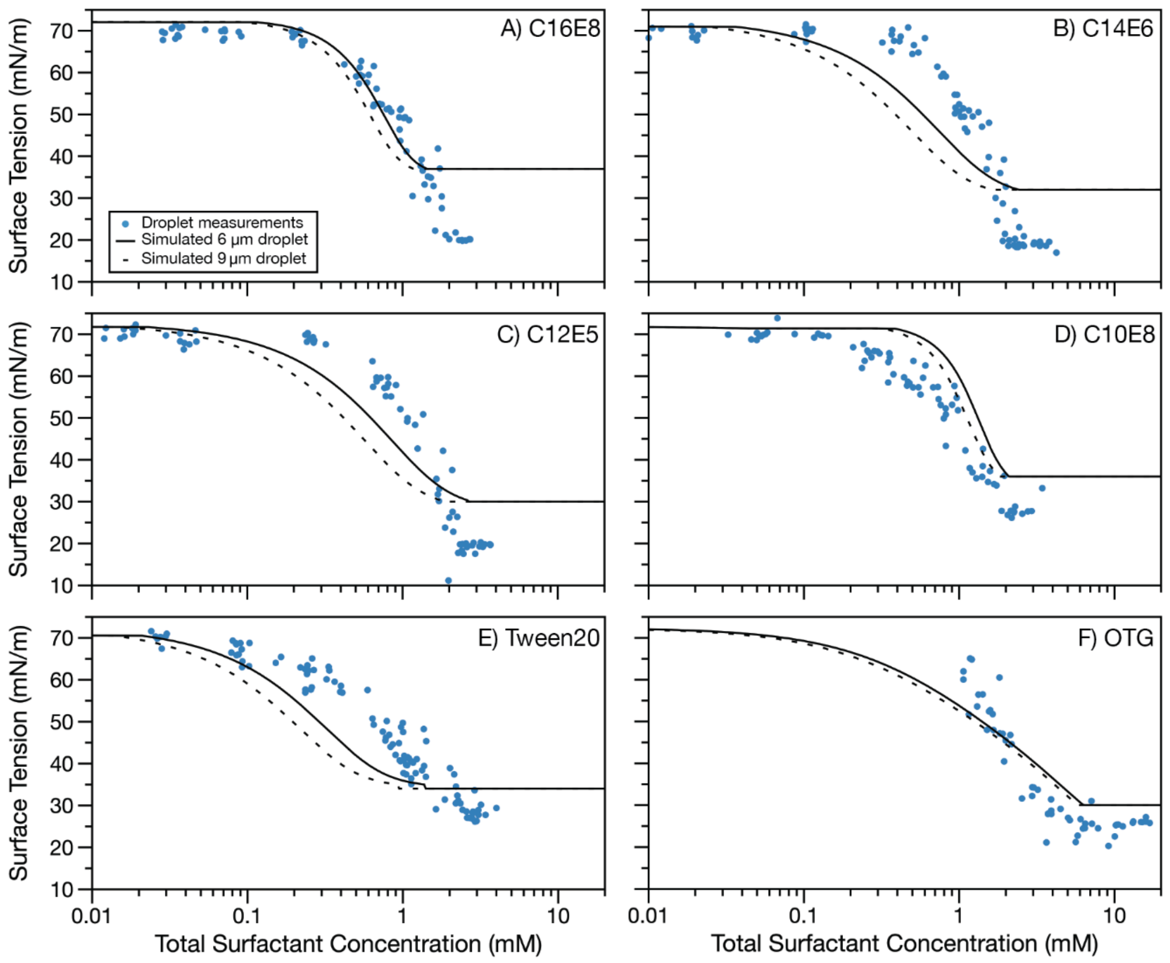

Fig. S2. Droplet surface tension measurements (blue points, composite droplet radius ≈ 6 – 9 μm) compared to simulated surface tension for 6 μm (solid black) and 9 μm (dashed black) radii. Droplets contain 0.5 M NaCl cosolute with (A) C16E8, (B) C14E6, (C) C12E5, (D) C10E8, (E) Tween20 and (F) OTG.

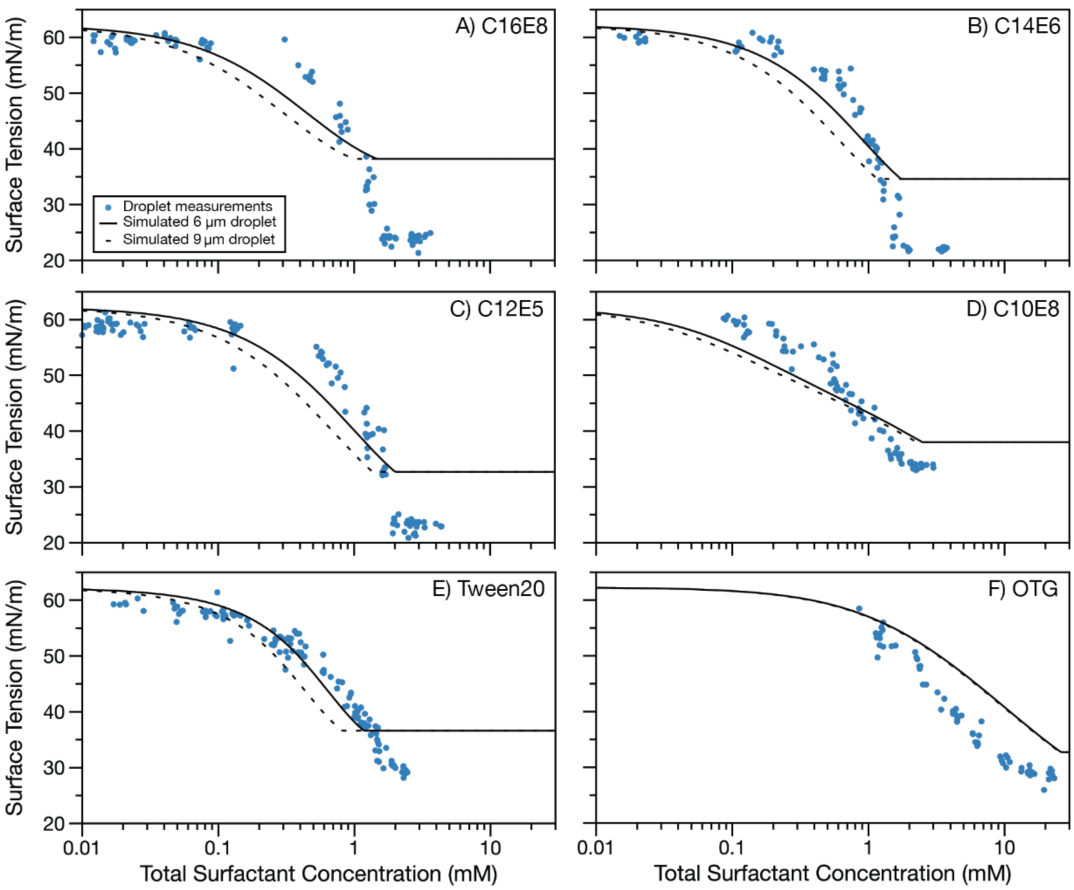

Fig. S3. Droplet surface tension measurements (blue points, composite droplet radius ≈ 6 – 9 μm) compared to simulated surface tension for 6 μm (solid black) and 9 μm (dashed black) radii. Droplets contain 0.9 M glutaric acid cosolute with (A) C16E8, (B) C14E6, (C) C12E5, (D) C10E8, (E) Tween20 and (F) OTG.

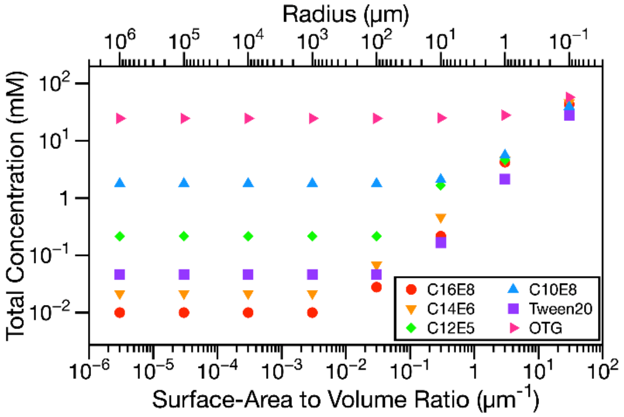

Fig. S4. MLMix predictions for total surfactant concentration required to reach a full monolayer surface coverage (and minimum surface tension) for 0.9 M glutaric acid and surfactant mixtures in 0.1 - 10^6 µm radius droplets.

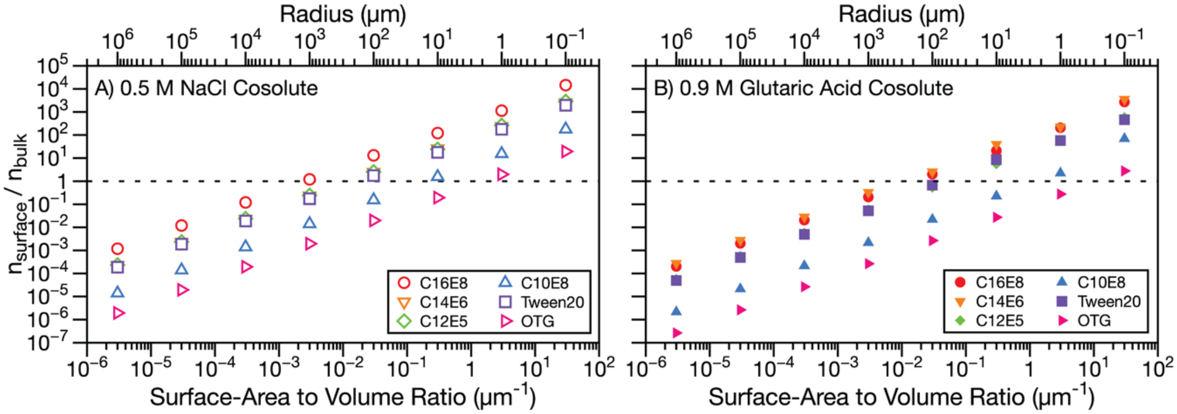

Fig. S5. Ratio of moles of surfactant at the surface to moles of surfactant in the bulk for droplets with radii spanning seven orders of magnitude for (A) 0.5 M NaCl cosolute and (B) 0.9 M glutaric acid cosolute. The black dashed line indicates a ratio of one, i.e. there are the same number of moles of surfactant at the droplet interface as in the droplet bulk.

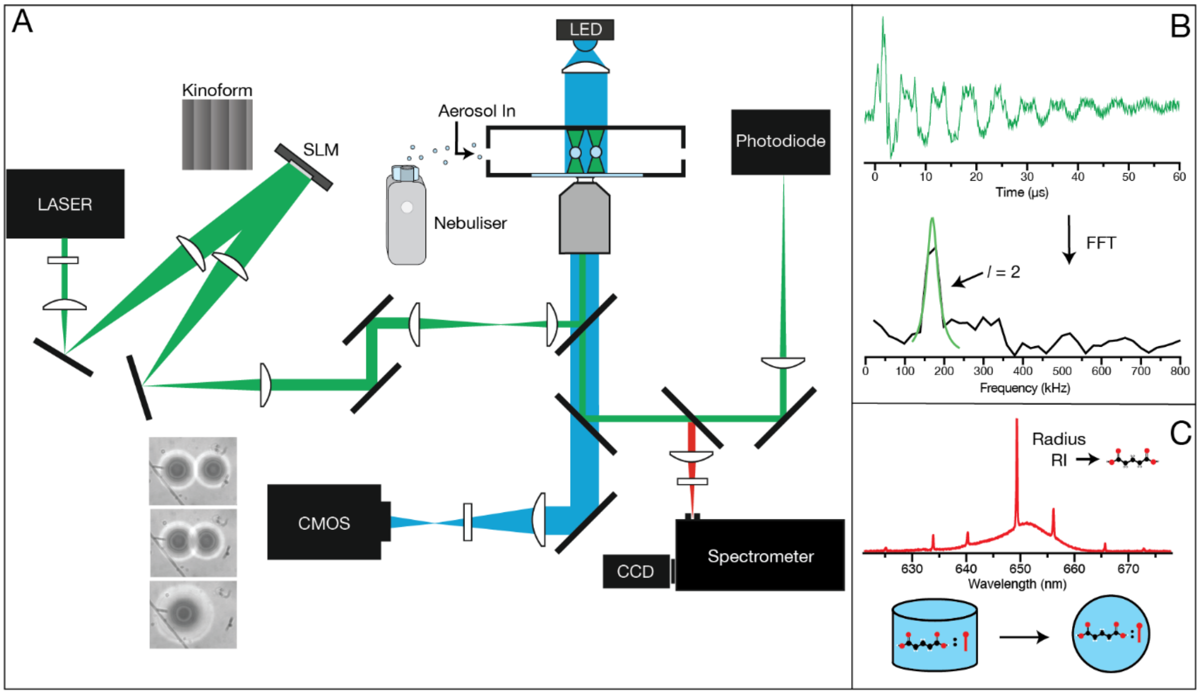

Fig. S6. Experimental set up and workflow. (**A**) Two optical traps are generated using a kinoform on a spatial light modulator (SLM). Once aerosol droplets are confined to the traps, they can be moved closer together until they meet and coalesce. (**B**) The elastically scattered light is collected with high time resolution with a photodiode and a fast-Fourier transform (FFT) of this trace provides the oscillation frequency of the surface modes. (**C**) Simultaneously, the cavity-enhanced Raman spectrum is collected with the spectrograph. The resonances in the spectrum are fit with Mie Theory to yield the radius and refractive index of the droplet resulting from coalescence. The concentration of cosolute is determined from the refractive index and the ratio of cosolute to total surfactant is assumed to be conserved between the macroscopic solution and the droplet.

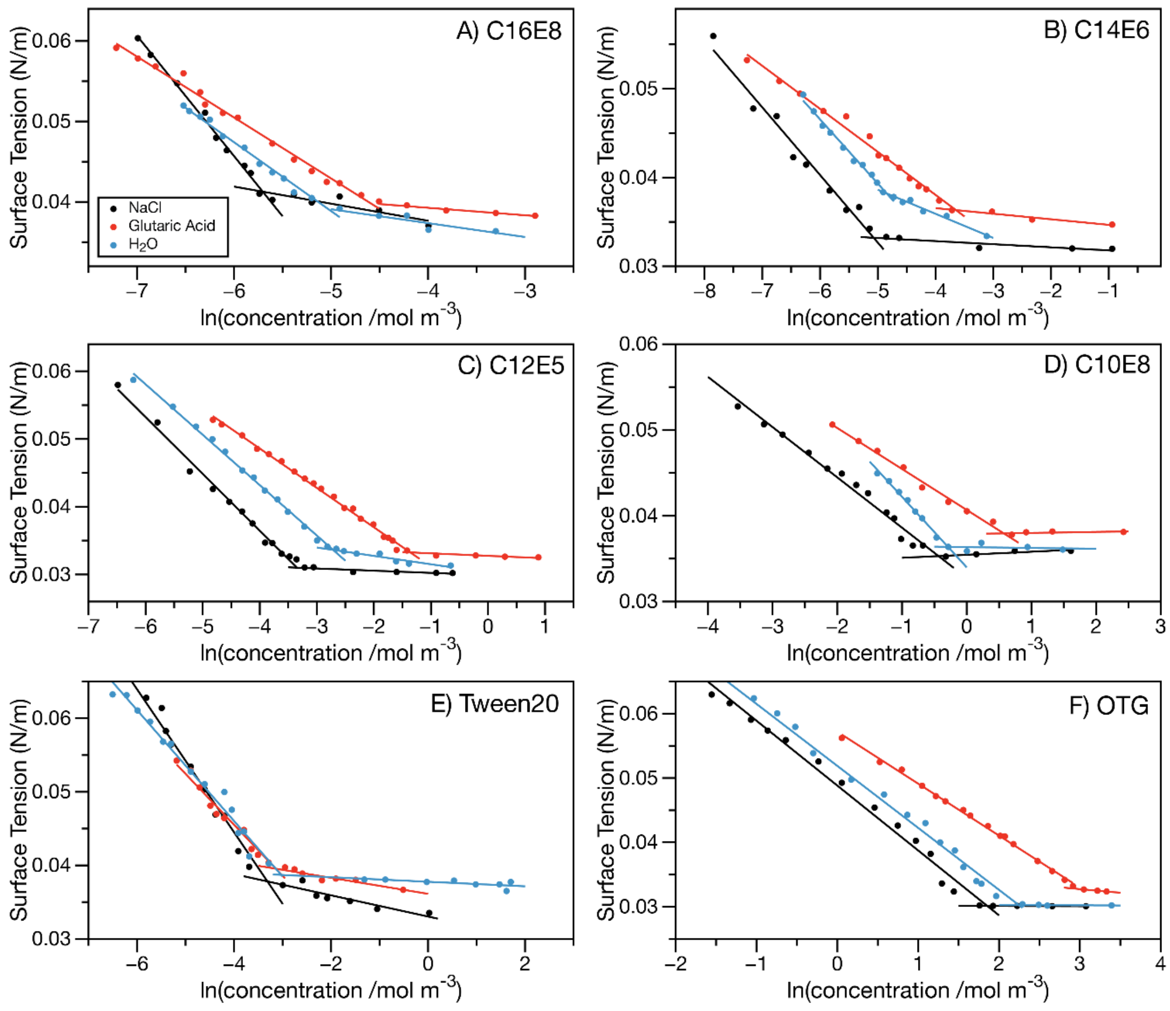

Fig. S7. Macroscopic surface tension measurements and isotherms fits. (A) C16E8, (B) C14E6, (C) C12E5, (D) C10E8, (E) Tween20 and (F) OTG. Each datapoint is an average of three repetitions. For each surfactant a dataset is shown for binary aqueous solution (blue) and ternary solutions with 0.9 M glutaric acid cosolute (red) as well as 0.5 M NaCl cosolute (black). Each dataset is fit with two straight lines, one in the region where increasing the surfactant concentration decreases the surface tension (the Gibbs isotherm) and one in the region where the surface tension plateaus at a minimum value. The CMC for each system is determined from the intersection of these two linear fits.

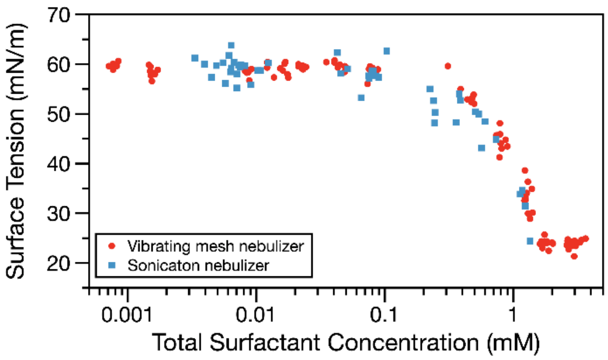

Fig. S8. Droplet surface tension as a function of total surfactant concentration for 0.9 M glutaric acid and C16E8 where droplets have been generated with a vibrating mesh nebulizer (red circles) and a sonication nebulizer (blue squares).

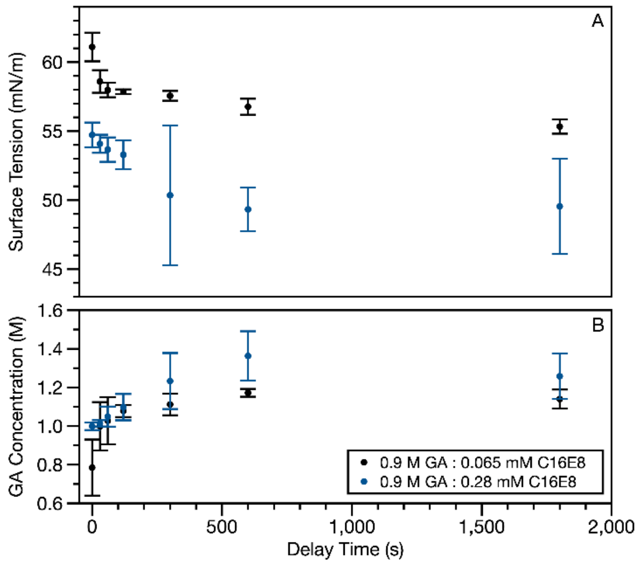

Fig. S9. (A) Droplet surface tension measured after a delay time and (B) the concentration of glutaric acid (GA) after a delay time determined by fitting the resonances in the cavity-enhanced Raman spectrum. Data points represent the average of at least three repeat measurements.

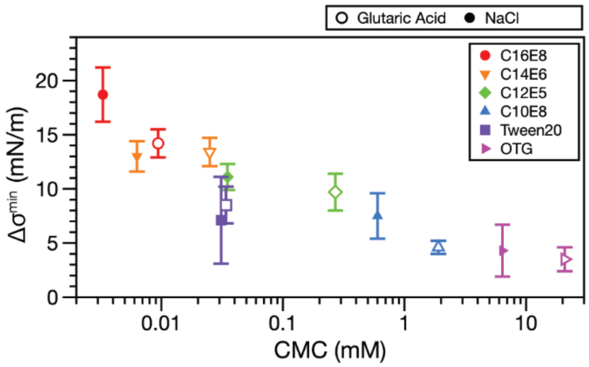

Fig. S10. Difference in minimum surface tension ($\Delta\boldsymbol{\sigma}^{\boldsymbol{min}} = \boldsymbol{\sigma}^{\boldsymbol{min}}_{\boldsymbol{macroscopic}} - \boldsymbol{\sigma}^{\boldsymbol{min}}_{\boldsymbol{droplet}}$) between droplet and macroscopic measurements. Surfactants with 0.9 M glutaric acid (open markers) and 0.5 M NaCl (filled markers) cosolutes as a function of CMC determined from the Gibbs isotherm analysis of macroscopic surface tension measurements. Error bars represent the maximum difference between the standard deviation of the mean for all surface tensions in the minimum region for the droplet and macroscopic measurements.

References

1. Bzdek, B. R., Power, R. M., Simpson, S. H., Reid, J. P. & Royall, C. P. Precise, contactless measurements of the surface tension of picolitre aerosol droplets. *Chem. Sci.* **7**, 274–285 (2016).
2. Bzdek, B. R., Reid, J. P., Malila, J. & Prisle, N. L. The surface tension of surfactant-containing, finite volume droplets. *Proc. Natl. Acad. Sci. U. S. A.* **117**, 8335–8343 (2020).
3. Preston, T. C. & Reid, J. P. Accurate and efficient determination of the radius, refractive index, and dispersion of weakly absorbing spherical particle using whispering gallery modes. *J. Opt. Soc. Am. B* **30**, 2113–2122 (2013).
4. Preston, T. C. & Reid, J. P. Determining the size and refractive index of microspheres using the mode assignments from Mie resonances. *J. Opt. Soc. Am. A* **32**, 2210–2217 (2015).
5. Bzdek, B. R., Collard, L., Sprittles, J. E., Hudson, A. J. & Reid, J. P. Dynamic measurements and simulations of airborne picolitre-droplet coalescence in holographic optical tweezers. *J. Chem. Phys.* **145**, 054502 (2016).
6. Song, Y. C. *et al.* Measurements and predictions of binary component aerosol particle viscosity. *J. Phys. Chem. A* **120**, 8123–8137 (2016).
7. *Handbook of Chemistry and Physics*. (CRC Press/Taylor & Francis, 2021).
8. Lin, S. Y., Lee, Y. C., Yang, M. W. & Liu, H. S. Surface equation of state of nonionic CmEn surfactants. *Langmuir* **19**, 3164–3171 (2003).
9. Mattei, M. & Kontogeorgis, G. M. Modeling of the critical micelle concentration (CMC) of nonionic surfactants with an extended group-contribution method. *Ind. Eng. Chem. Res.* **52**, 12236–12246 (2013).
10. Yan, C., Angus-Smyth, A. & Bain, C. D. Adsorption kinetics of non-ionic surfactants in micellar solutions: Effects of added charge. *Faraday Discuss.* **160**, 45–61 (2013).
11. Theander, K. & Pugh, R. J. Synergism and foaming properties in mixed nonionic/fatty acid soap surfactant systems. *J. Colloid Interface Sci.* **267**, 9–17 (2003).
12. Binks, B. P., Fletcher, P. D. I., Paunov, V. N. & Segal, D. Equilibrium and dynamic adsorption of C12E5 at the air-water surface investigated using ellipsometry and tensiometry. *Langmuir* **16**, 8926–8931 (2000).
13. Shah, V., Bharatiya, B., Shukla, A. D., Mukherjee, T. & Shah, D. O. Adsorption of nonionic Brij and Tween surfactants at PTFE-water and air-water interfaces: Investigations on wetting, dispersion stability, foaming and drug solubilization. *Colloids Surfaces A Physicochem. Eng. Asp.* **508**, 159–166 (2016).
14. Tran, C. D. & Yu, S. Near-infrared spectroscopic method for the sensitive and direct determination of aggregations of surfactants in various media. *J. Colloid Interface Sci.* **283**, 613–618 (2005).
15. Bakshi, M. S., Kaura, A. & Mahajan, R. K. Effect of temperature on the micellar properties of polyoxyethylene glycol ethers and twin tail alkylammonium surfactants. *Colloids Surfaces A Physicochem. Eng. Asp.* **262**, 168–174 (2005).
16. Molina-Bolívar, J. A., Aguiar, J., Peula-García, J. M. & Ruiz, C. C. Surface activity, micelle formation, and growth of n-octyl-β-D-thioglucopyranoside in aqueous solutions at different temperatures. *J. Phys. Chem. B* **108**, 12813–12820 (2004).
17. Frindi, M., Michels, B. & Zana, R. Ultrasonic absorption studies of surfactant exchange

between micelles and bulk phase in aqueous micellar solutions of nonionic surfactants with a short alkyl chain. 3. Surfactants with a sugar head group. *J. Phys. Chem.* **96**, 8137–8141 (1992).

18. Rusdi, M., Moroi, Y., Hlaing, T. & Matsuoka, K. Micelle formation and surface adsorption of octaethylene glycol monoalkyl ether (CnE8). *Bull. Chem. Soc. Jpn.* **78**, 604–610 (2005).
19. Malila, J. & Prisle, N. L. A monolayer partitioning scheme for droplets of surfactant solutions. *J. Adv. Model. Earth Syst.* **10**, 3233–3251 (2018).
20. Gaman, A. I. *et al.* Binary homogeneous nucleation in water-succinic acid and water-glutaric acid systems. *J. Chem. Phys.* **120**, 282–291 (2004).
21. Vanhanen, J. *et al.* Ternary solution of sodium chloride, succinic acid and water; Surface tension and its influence on cloud droplet activation. *Atmos. Chem. Phys.* **8**, 4595–4604 (2008).
22. Janz, G. J. Molten salts data as reference standards for density, surface tension, viscosity, and electrical conductance: KNO3 and NaCl. *J. Phys. Chem. Ref. Data* **9**, 791–830 (1980).
23. Kodama, M. & Miura, M. The second CMC of the aqueous solution of sodium dodecyl sulfate. II. Viscosity and density. *Bull. Chem. Soc. Jpn.* **45**, 2265–2269 (1972).
24. Rowe, A. M. & Chou, J. C. S. Pressure-volume-Temperature-concentration relation of aqueous NaCl solutions. *J. Chem. Eng. Data* **15**, 61–66 (1970).
25. Cai, C. *et al.* Comparison of methods for predicting the compositional dependence of the density and refractive index of organic-aqueous aerosols. *J. Phys. Chem. A* **120**, 6604–6617 (2016).
26. Alvarez, N. J., Walker, M. & Anna, S. L. A criterion to assess the impact of confined volumes on surfactant transport to liquid – fluid interfaces. *Soft Matter* **8**, 8917–8925 (2012).
27. Lyubimov, D. V., Konovalov, V. V., Lyubimova, T. P. & Egry, I. Small amplitude shape oscillations of a spherical liquid drop with surface viscosity. *J. Fluid Mech.* **677**, 204–217 (2011).
28. Wolk, J. & Strey, R. Homogeneous nucleation of H2O and D2O in comparison: The isotope effect. *J. Phys. Chem. B* **105**, 11683–11701 (2001).
